# Public social protection policies for people affected by tuberculosis: a documentary analysis*

**DOI:** 10.1590/1518-8345.7526.4503

**Published:** 2025-03-14

**Authors:** Melisane Regina Lima Ferreira, Jaqueline Garcia de Almeida Ballestero, Rubia Laine de Paula Andrade, Tiemi Arakawa, Inês Fronteira, Aline Aparecida Monroe

**Affiliations:** 1 Universidade de São Paulo, Escola de Enfermagem de Ribeirão Preto, PAHO/WHO Collaborating Centre for Nursing Research Development, Ribeirão Preto, SP, Brazil; 2 Scholarship holder at the Coordenação de Aperfeiçoamento de Pessoal de Nível Superior (CAPES), Brazil; 3 Ministério da Saúde, Coordenação Geral de Vigilância da Tuberculose, Micoses endêmicas e Micobactérias não tuberculosas, Departamento de HIV/Aids, Tuberculose, Hepatites Virais e Infecções Sexualmente Transmissíveis, Brasília, DF, Brazil; 4 Universidade NOVA de Lisboa, Escola Nacional de Saúde Pública, Lisboa, Portugal; 5 Scholarship holder at the Conselho Nacional de Desenvolvimento Científico e Tecnológico (CNPq), Brazil

**Keywords:** Tuberculosis, Public Policy, Social Welfare, Health Policy, Human Rights, Social Security

## Abstract

to analyze the normative documents that seek to guarantee the right to social protection for people affected by tuberculosis in force in Brazil in 2023.

qualitative documentary research carried out in September 2023, based on the survey of documents at the national, state and municipal levels, from government agencies and social control bodies after the promulgation of the Federal Constitution, on four electronic platforms, exported and organized in the Atlas.ti software, and interpreted based on content analysis, thematic mode.

the analytical *corpus* consisted of 30 normative documents — nine laws, seven technical-institutional materials, five ordinances, four resolutions, two decrees, a technical cooperation agreement, a normative instruction, and an operational instruction — from which four thematic categories emerged: the right to health care, the right to social care, the right to social security, and the sharing of responsibilities.

policies to protect people with tuberculosis in Brazil are recent and there is still much room for improvement toward a comprehensive approach through intersectoral and interministerial coordination, aiming to address social vulnerability and reaffirming the State’s duty to guarantee social protection by means of public policies that promote life, citizenship, human rights, and social justice.

## Highlights

**(1)** The right to health care includes public policies in the scope of SUS to address TB. **(2)** The right to social care emphasized access to services, programs, and benefits. **(3)** The right to social security covered retirements and benefits for people with TB. **(4)** Interministerial responsibility, intersectoral responsibility, and responsibility shared between SUS and SUAS. **(5)** Challenges in accessing rights to social care and health care persist for people with TB.

## Introduction

Tuberculosis (TB) is one of the most emblematic diseases related to poverty and strongly permeated with social determinants. At the same time, it is considered that it perpetuates cycles of misery due to the social and economic impacts resulting from illness^(1)^. As a multi-causal disease, TB requires a multisectoral response and synergism between public social protection policies that seek to eliminate poverty and promote equity, justice and human rights for people affected by TB, including addressing all forms of discrimination and stigmatization^(2)^.

The World Health Organization’s (WHO) End TB Strategy recommends reducing incidence rates by 90% and mortality rates by 95% by 2035 to eliminate it as endemic by 2050, with the additional goal that no person with TB needs to bear catastrophic costs or social repercussions due to the disease^(3)^. Consistently, in line with the United Nations Sustainable Development Goals (SDGs), TB is included in Target 3.3 of SDG 3, whose proposal is to reduce TB deaths by 90% and new TB cases by 80% by 2030. This goal is also closely related to the tenth goal regarding the reduction of inequalities^(4)^.

However, in 2022, TB was estimated to have affected 10.6 million people worldwide, with 1.3 million deaths from the disease and 167,000 deaths among people living with HIV/AIDS. In the same year, the Americas region had 3.1% of the total TB cases worldwide and much higher estimates of deaths caused by the disease than in 2015 (+41%)^(3)^. In this region, only Brazil ranks among the 30 countries with the highest TB burdens in the world, since, in 2022, the country had 78,057 new TB cases, with an incidence rate of 36.3 cases/100,000 inhabitants, in addition to 5,162 deaths from the disease, with a mortality rate of 2.2 deaths/100,000 inhabitants^(5)^.

This means that the country is still far from meeting the proposals of the End TB Strategy and of the National Plan to End TB as a public health issue, which recommend reducing the TB incidence rate to less than 10 cases/100,000 inhabitants and one death/100,000 inhabitants by 2035^(6-7)^. Considering this situation, one of the boldest proposals to end TB is based on the 2nd Pillar referring to bold policies and support systems, aiming at the social protection of people affected by the disease, in addition to the reduction of poverty and other determining factors for TB disease^(3)^.

Social protection refers to actions that involve public policies geared toward guaranteeing life and human rights, preventing the incidence of risks and vulnerabilities, reducing damages and negative impacts due to social, economic, political, natural restrictions or offenses against human dignity^(8)^. In order to address TB, a literature review conducted from a global perspective showed that measures and strategies oriented toward social protection as a right of people affected by TB improve nutritional status, quality of life, and adherence to treatment, reducing catastrophic costs and promoting favorable treatment results^(9)^.

In addition, a study that globally analyzed the association between social protection spending and TB burden showed that countries that invest a large portion of their Gross Domestic Product (GDP) in social protection policies see lower TB prevalence, incidence and mortality rates^(10)^. Such issues demonstrate the alignment of social protection with public policies proposed to overcome the challenge of eliminating TB as a public health problem at the national and international levels.

However, there are still gaps in the understanding of how social protection measures and strategies are regulated as public policies, and how they are oriented towards implementation in different socioeconomic contexts. Accordingly, this study seeks to contribute to the advancement of scientific knowledge by examining how protection policies for people with TB are regulated at the national level and how they can be adapted in a more inclusive manner to improve the support and treatment mechanisms available, impacting the quality of life of people affected by TB and the efficiency of health care systems.

In this sense, public policies aimed at overcoming inequalities that affect human health and are characterized as unjust are fundamental to achieve a more equitable society and, therefore, impact TB control^(11)^. Therefore, a conjunctural analysis is opportune, considering the potential of Brazil and the future challenges that the financial crisis and austerity policies may pose for the Brazilian health care and social care systems.

Thus, guided by the research question — “How is social protection for people affected by TB included in the framework of Brazilian public policies?” —, the objective was to analyze normative documents that seek to guarantee the right to social protection for people affected by TB in force in Brazil in 2023.

## Method

### Study design

This is a qualitative documentary research of documents that regulate public policies that include in their scope social protection measures for people affected by TB in the Brazilian national level. This type of research is characterized by the search for information in documents with original data based on methods and techniques for apprehension, understanding and analysis of factual information by the researcher, since the documents underwent no prior scientific treatment^(12-13)^.

The use of documents in research provides much recovered information that enables expanding the understanding of issues whose understanding requires a historical and sociocultural contextualization^(12)^. In this sense, official documents are considered reliable data sources, as they allow a contextual analysis of normative and political acts^(14)^.

### Selection criteria

We opted for the inclusion of documents subsequent to the historical-temporal framework of the 1988 Federal Constitution, which guarantees social protection in Brazil through health care, social care and social security policies, which constitute social security in the country^(15)^. Based on this context, we surveyed institutional technical materials, as well as laws, decrees, ordinances, resolutions and other national, state or municipal normative acts, from government agencies and their social control bodies, that are in force.

We excluded documents related to the incorporation of new anti-tuberculosis therapeutic regimens, diagnostic tests for TB, public consultations, bills, documents instituted by international bodies, as well as regulations that did not specify social protection measures for people affected by TB.

### Documentary survey

The searches were performed by the main researcher, in September 2023, on the following electronic platforms: Official Federal Gazette (DOU) (https://www.in.gov.br/consulta/) for federal, state and/or municipal documents; Portal of Brazilian Federal Legislation (https://legislacao.presidencia.gov.br/) for federal documents; Portal of State Laws (https://leisestaduais.com.br/) for state and/or municipal documents; and official website of publications of the Ministry of Health (https://www.gov.br/saude/pt-br/centrais-de-conteudo/publicacoes) for federal documents, using the keywords: “Tuberculosis” and “Social Protection.”

For the searches in the DOU, in order to optimize the documentary survey process, the “Main Organization” filter was used to select documents issued by bodies with direct competence over social protection policies and the “Type of Act” filter was used to select specific acts that establish or regulate policies and practices related to social protection, with normative and legal relevance. In addition, to search for documents with dates prior to Jan 1, 2018, we used the “Search in the Certified Version” or “Full Certified Gazzette” filters. For the Ministry of Health website, we used the “all publications” filter, and for the other platforms we used the filters for “standards in force” or “published” and time frame from 1988.

It should be noted that, to reduce a possible bias of interest in the use of the Portal of State Laws, we adopted measures for transparency in the choice of state and municipal legislation and a rigorous data analysis to avoid distortions and preserve the integrity and impartiality of this study. It is also noted that documents from organized civil society websites were not included, since the objective of the study was related to examining the policies implemented and not the demands of the different social actors.

### Data processing and analysis

After the survey and access to official publications, the documents were saved in PDF format and exported to the Atlas.ti software, version 23, to be organized and interpreted using content analysis in thematic mode, which was conducted in three stages according to the assumptions of Bardin^(16)^, namely: documentary pre-analysis; exploration of material; and treatment of results. In the pre-analysis stage — conducted entirely by two researchers, including the main one —, a floating reading of the texts was carried out in order to trace their relation with the study objective and formulate cores of meaning based on five dimensions^(14)^: analysis of context; authorship; interests and/or reliability of the text; nature of the text; and key concepts of the text.

Then, the material was explored through an in-depth reading of the documents that constituted the analytical *corpus*. The main researcher extracted information from the documents using a Microsoft Excel spreadsheet for codification and categorization according to type, year of publication, body or institution of origin, scope, and topics and/or themes addressed. These categories were certified by two other researchers, whose certification stage permeated the review and validation of the categories in relation to the data extracted and to compliance with the study object.

In the treatment of the results stage, we performed simple descriptive statistics of data related to document categorization. Finally, the findings were interpreted by the three researchers directly involved in the previous stages (including the main one), according to the study questioning to be analyzed from the perspective of the right to social protection, which covers measures oriented toward guaranteeing health care, social care, and social security. It is noted that this study is based on an expanded conception of health proposed by the Brazilian Health Reform movement and on the notion of right as a social achievement, with the State being responsible for providing the indispensable conditions for its full exercise and ensure universal and equal access to health promotion, protection and recovery actions and services^(17)^.

### Ethical aspects

Although ethical approval is not required for documentary research, it should be noted that this study is part of a research on the assessment of social protection for people affected by TB, which was approved by the Research Ethics Committee of the institution in charge under opinion No. 6,389,278, of Oct 5, 2023 (CAAE 71246023.6.0000.5393). In addition, we tried to use non-stigmatizing language related to TB throughout the study^(18)^.

## Results

**Figure 1 -:**
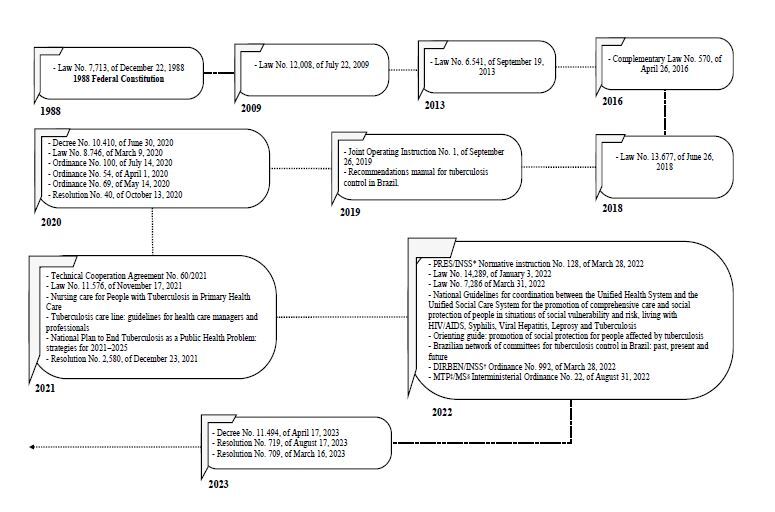
Historical timeline representing the paths of public social protection policies for people affected by TB in Brazil

The documentary survey on the electronic platforms selected in this study resulted in the inclusion of 30 normative documents published through nine laws (30%)^(19-27)^, seven technical-institutional materials (23.4%)^(6,28-33)^, five ordinances (16.7%)^(34-38)^, four resolutions (13.3%)^(39-42)^, two decrees (6.7%)^(43-44)^, one technical cooperation agreement (3.3%)^(45)^, one normative instruction (3.3%)^(46)^ and one operational instruction (3.3%)^(47)^, in the period between 1988 and August 2023, with the year 2022 having the highest number of publications (n=8, 26.6%) (Figure 1).

*PRES/INSS = Legal Medicine of the National Institute of Social Security; ^†^DIRBEN/INSS = Directorate of Benefits of the National Institute of Social Security; ^‡^MTP = Ministry of Labor and Social Security; ^§^MS = Ministry of Health

The included regulations that regulate or guide social protection for people affected by TB (Figure 2) came mostly from government bodies of national scope (n=22, 73.3%), such as the Chief of Staff of the Presidency^(21-22,26-27,43-44)^, the Ministry of Human Rights and Citizenship^(36-39,45,47)^, the Ministry of Health^(28-33)^ and the Ministry of Labor and Employment^(34-35,46)^; state scope (n=5, 16.7%), such as the Legislative Assembly of Mato Grosso^(20)^ and state governments of Rio de Janeiro^(23,25,41)^ and Rio Grande do Norte^(19)^; and municipal scope (n=1, 3.3%), through the Municipal Council of Rio de Janeiro^(24)^. In addition, two publications (6.7%) came from the National Health Council^(40,42)^.

**Figure 2 -:** Characterization of normative documents that seek to guarantee the right to social protection for people affected by TB, as to their name, definitions and relation with social protection for people affected by TB (n = 30)

**Document name**	**Definitions and coordination with social protection for people affected by TB**
Technical Cooperation Agreement No. 60/2021 ^( 45 )^	- Integration of public health policies into social care;
	- Social benefits for people in situation of social vulnerability and more susceptible to tuberculosis;
	- Development of specific programs that address the needs of people with tuberculosis.
Decree No. 10,410, of June 30, 2020 ^( 43 )^	- Adjusts the rules for access to social security benefits, such as sick pay, disability retirement and other benefits that are essential for workers who are temporarily or permanently disabled due to tuberculosis.
	- Contributes to the prevention of socioeconomic vulnerability of people affected by tuberculosis.
Decree No. 11.494, of April 17, 2023 ^( 44 )^	- Establishment of a committee that coordinates inter-ministerial efforts to address the social determinants of tuberculosis;
	- Integration and implementation of policies that protect the rights and well-being of people with tuberculosis.
PRES/INSS* Normative Instruction No. 128, of March 28, 2022 ^( 46 )^	- Establishes clear and accessible procedures for access to social security benefits, ensuring that people with TB receive the necessary financial support during treatment and recovery.
	- Provides a financial safety net for people with TB and helps mitigate the economic impact of the disease.
Joint Operating Instruction No. 1, of September 26, 2019 ^( 47 )^	- Promotes collaboration between health care and social care that is necessary to overcome the barriers imposed by tuberculosis, such as loss of income, need for food support, other vulnerabilities and social exclusion.
Complementary Law No. 570, of April 26, 2016 ^( 19 )^	- Strengthens the social protection for people with tuberculosis by ensuring access to disability retirement with full benefits, providing financial security and recognizing the severity of the disease in legal and social security terms.
Law No. 11.576, of November 17, 2021 ^( 20 )^	- Creates a specific state policy for the control and elimination of tuberculosis through coordination with other areas, such as social care, housing and food, which are fundamental for the social protection of affected people.
Law No. 13.677, of June 13, 2018 ^( 21 )^	- Provides access to financial resources that can be used to meet emerging needs during tuberculosis treatment and afford financial relief, supplementing other forms of social and economic support.
Law No. 14.289, of January 3, 2022 ^( 22 )^	- Promotes social protection by preventing tuberculosis-related stigmatization and discrimination by guaranteeing human rights and dignity and creating a safer, more confidential environment so people can seek and continue treatment without fear of undue exposure.
Law No. 6.541, of September 19, 2013 ^( 23 )^	- Contributes to social protection by facilitating access to treatment, reducing costs associated with the disease, encouraging continuity of treatment and promoting social inclusion of people with tuberculosis.
Law No. 7,286, of March 31, 2022 ^( 24 )^	- Creates a specific municipal policy for the control and elimination of tuberculosis through coordination with other areas, such as social care, housing and food security, which are fundamental for the social protection of affected people.
Law No. 8.746, of March 9, 2020 ^( 25 )^	- Creates a specific state policy for the control and elimination of tuberculosis through guarantee of health care services, coordination of public policies and protection against social discrimination and exclusion.
Law No. 12.008, of July 29, 2009 ^( 26 )^	- Contributes to the social protection of people with TB by facilitating quick and efficient access to rights and benefits, reducing bureaucracy and delays, and providing additional protection and adequate legal support for those facing the disease.
Law No. 7.713, of December 22, 1988 ^( 27 )^	- By allowing tax exemptions and deductions for people affected by tuberculosis, it can reduce the financial burden, alleviate the costs associated with treatment and promote crucial economic support for the management of the disease.
Nursing care for people with tuberculosis in Primary Health Care ^( 28 )^	- In association with social protection by ensuring access to adequate health care, promote education, carry out continuous monitoring, identify and refer people with tuberculosis to specialized treatment, provide psychosocial support, and coordinate with other services.
National Guidelines for coordination between the Unified Health System and the Unified Social Care System to promote comprehensive health care and social protection for people in situation of social vulnerability and risk, living with HIV/AIDS, Syphilis, Viral Hepatitis, Leprosy and Tuberculosis ^( 29 )^	- Establishes a framework for the integration of health care and social care;
	- Promotes comprehensive health care and ensures that people with TB receive the support they need to meet their complex needs and improve their quality of life.
	- Addresses additional vulnerabilities such as financial problems, stigmatization and lack of access to services.
Orienting guide: promotion of social protection for people affected by tuberculosis ^( 30 )^	- Provides guidelines so people with TB receive comprehensive and effective support, addressing their health and social needs and promoting an integrated social protection system;
	- Offers information on programs and policies geared toward reducing the financial burden for people with TB and their family members.
Tuberculosis care line: guidelines for health care managers and professionals ^( 31 )^	- Provides guidelines for coordinated, accessible and high-quality health care, in addition to offering additional support that improves treatment adherence and quality of life for people with tuberculosis.
Manual of Recommendations for Tuberculosis Control in Brazil ^( 32 )^	- Establishes guidelines related to social protection through effective care integrated between health care and social care;
	- Provides guidelines for the education and training of health care professionals and ensures that people with tuberculosis have access to the resources and support necessary to face the disease and its consequences.
National Plan to End Tuberculosis as a Public Health Problem: strategies for 2021–2025 ^( 6 )^	- Establishes a strategic approach integrated between health care policies and social protection policies to control the disease;
	- Recommends actions for equitable access to treatment, reducing financial costs and improving social support and resources available to people with TB.
Brazilian network of committees for tuberculosis control in Brazil: past, present and future ^( 33 )^	- Addresses the coordination of efforts between different entities and bodies of government for the development of social protection policies and practices;
	- Well-structured and operational committees help ensure that people with TB have access to the health care, social care and resources they need to face the disease.
^†^ DIRBEN/INSS Ordinance No. 992, of March 28, 2022 ^( 34 )^	- Regulates access to social security benefits, such as disability retirement or sick pay;
	- Guarantees that people with TB receive the necessary financial support to offset the loss of income due to inability to work.
MTP ^‡^ /MS ^§^ Interministerial Ordinance No. 22, of August 31, 2022 ^( 35 )^	- Defines guidelines for collaboration between ministries responsible for health and labor, ensuring a coordinated approach that addresses health needs, employment issues and social protection in an integrated and effective manner for people with TB.
Ordinance No. 100, of July 14, 2020 ^( 36 )^	- Ensures that essential social care services continue to be provided during public health emergencies, ensuring continuity of care and adaptation of services to new conditions, promoting support for both tuberculosis management and comprehensive social protection.
Ordinance No. 54, of April 1, 2020 ^( 37 )^	- Ensures the continuity of social care services during public health crisis contexts, focusing on safety and health measures for users and professionals, ensuring that people with tuberculosis receive critical services for social protection, such as financial assistance and social support, without compromising their health.
Ordinance No. 69, of May 14, 2020 ^( 38 )^	- Addresses the circumstance of the homeless population during public health emergencies, ensuring that these people, who often face additional vulnerability conditions, are socially protected and receive necessary care for the management of their health conditions, including tuberculosis.
Resolution No. 40, of October 13, 2020 ^( 39 )^	- Ensures that the human rights and specific needs of the homeless population affected by tuberculosis are met in an integrated and comprehensive manner through public policies aligned to protect them.
Resolution No. 719, of August 17, 2023 ^( 40 )^	- Defines guidelines and proposals that can directly impact health care and social care policies to improve prevention, treatment and support for people with TB.
Resolution No. 2,580, of December 23, 2021 ^( 41 )^	- Ensures the allocation of resources for specific actions aimed at improving the care and support for people with tuberculosis;
	- Fosters collaboration between health care and social care and strengthens the capacity of municipalities, promoting more robust social protection for the affected population.
Resolution No. 709, of March 16, 2023 ^( 42 )^	- Contributes to a more effective and holistic social protection system, ensuring that people with TB receive the necessary support to overcome the disease and improve their quality of life, through the integration of health care and social care policies.

*PRES/INSS = Legal Medicine of the National Institute of Social Security; ^†^DIRBEN/INSS = Directorate of Benefits of the National Institute of Social Security; ^‡^MTP = Ministry of Labor and Social Security; ^§^MS = Ministry of Health

The documentary analysis led to the emergence of four thematic categories that covered their respective units of meaning and the main results traced in the normative documents included as part of the study *corpus* (Figure 3). The first thematic category, the *right to health*, refers to documents that presented social and economic policies to guarantee TB prevention and care actions in the Unified Health System (SUS), food and nutrition security, transportation, work, social control, and prevention of TB stigmatization and discrimination. It is noted that, in this category, we also traced documents specifically aimed at the social protection of homeless people affected by TB.

The second thematic category, the *right to social care*, included documents that presented access to social care services, programs, projects and benefits, the right to the Continuous Cash Benefit (BPC), eventual benefits, civil documentation, income transfer programs, and care services of the Unified Social Care System (SUAS). The *right to social security* was the third thematic category that covered documents that presented the right of people affected by TB to retirement due to permanent disability, temporary disability benefit (sick pay), exemption of income tax and financial transactions linked to the Social Integration Program and the Civil Servant Asset Formation Program (PIS/PASEP).

The last thematic category included documents for the *sharing of responsibilities* in addressing TB, through the work of SUAS in coordination with the SUS, intersectoral and interministerial actions, and regulations oriented toward speeding up justice for people affected by TB.

**Figure 3 -:** Description of the documentary analysis as to the four thematic categories, units of meaning and main results found in the normative documents that seek to guarantee the right to social protection for people affected by tuberculosis (n = 30)

**Thematic categories**	**Units of meaning**	**Main results (normative documents)**
*Right to Health Care*	Tuberculosis prevention and care actions in SUS*	Comprehensive care for health, economic, psychological and social needs of people affected by tuberculosis ^( 20 , 24 - 25 )^ , through referrals to other public policies ^( 47 )^ .
		Consolidation and strengthening of the tuberculosis care line, in order to guarantee access to care and its continuity between different services and levels of health care ^( 42 )^ .
		Timely identification of people with signs and symptoms of tuberculosis by health care and social care teams ^( 30 )^ .
		Strengthening and expansion of health promotion actions by Primary Health Care teams and programs, with emphasis on the Family Health Strategy ^( 39 )^ .
		Coordination of the SUS* Single Therapeutic Project and the SUAS ^†^ Individual Care Plan for comprehensive care of people with tuberculosis and their families ^( 47 )^ .
		*Homeless population affected by tuberculosis*: coordination and sharing of care between the Pop Center ^‡^ teams and the Street Clinics health teams ^( 37 , 39 )^ .
	Food and nutrition security	Guarantee of healthy and adequate nutrition ^( 24 - 25 , 29 , 40 )^ through incentives such as snacks ^( 28 )^ , milk ^( 28 )^ , provision of basic food baskets ^( 25 , 28 - 29 , 33 , 41 )^ , food vouchers or food cards ^( 41 )^ .
		Regular and permanent access to food with sufficient quality and quantity, through the use of popular restaurants ^( 25 , 29 - 30 , 32 , 41 )^ , food banks, community kitchens, popular fairs and markets ^( 30 )^ and integration with the National Food and Nutrition Security System ^( 47 )^ .
	Transportation	Exemption from payment of tariffs in intercity transport services ^( 23 , 25 , 30 , 32 )^ , by granting a maximum of 60 social vouchers monthly within 30 business days ^( 23 )^ or within 15 business days ^( 25 )^ .
		Guarantee of the social voucher for the companion of the person with tuberculosis who cannot move around unaccompanied ^( 25 , 32 )^ or who is unable to move around alone ^( 32 )^ .
		Access to free transportation ^( 29 )^ through the provision of transportation vouchers ^( 28 )^ on ferries, subways, buses and trains ^( 32 )^ .
	Work	Programs to promote access to the world of work ^( 29 )^ .
		Maintenance of work relations through the prohibition of dismissal from work for having contracted tuberculosis, guarantee of healthy settings and withdrawal from the FGTS ^§(30)^ .
	Social control	Encouragement to the participation of civil society representatives, community representatives and leaders, and people affected by tuberculosis in the social control bodies of SUS* and SUAS ^†(29-30,33,45)^ .
		Establishment of forums for coordination between social care and health care ^( 45 )^ .
	Prevention of tuberculosis stigmatization and discrimination	Promotion of actions for social inclusion of people with tuberculosis ^( 30 , 45 )^ , within the scope of health care and social care units, in the territory and in community spaces ^( 6 , 47 )^ .
		Prohibition of submission to inhuman or degrading treatment, deprivation of liberty or family life, and discrimination due to morbidity ^( 20 , 25 )^ .
		Obligation to keep the confidentiality about the condition of people with tuberculosis ^( 22 )^ .
		Information on discriminatory situations, on the use of channels for reporting and filing complaints through ombudsperson offices or services such as “Dial 100,” “Dial 180,” and “Dial Health 136” ^( 29 )^ .
*Right to Social Care*	Access to social care services, programs, projects and benefits	Guarantee of access and inclusion in the Single Registry (CadÚnico) ^( 28 , 30 , 32 , 47 )^ for the qualified offer of social care services, programs, projects and benefits of the federal government to people with tuberculosis in a situation of risk and vulnerability ^( 6 , 28 , 31 , 45 , 47 )^ .
		Recognition of people with tuberculosis as meeting eligibility criterion for social care programs and services ^( 29 , 32 )^ .
		Use of CRAS ^||^ , CREAS ^¶^ , Pop Center ^‡^ , and Care Units, among others, as spaces for activities oriented toward the social protection of people affected by tuberculosis ^( 28 - 29 , 47 )^ .
	BPC**	Provision of a monthly minimum wage for people with disabilities of any age or the elderly, aged 65 years or older, who are unable to support themselves or be supported by the family ^( 30 , 32 )^ .
	Eventual benefits	Access to supplementary and temporary financial benefits in situations of birth, death, provisional vulnerability and public calamity ^( 30 , 32 )^ .
	Civil documentation	Access to basic civil documentation ^( 29 - 30 , 47 )^ .
	Income transfer programs	Recognition of people with tuberculosis as meeting eligibility criterion for income transfer programs ^( 29 )^ , such as PBF ^††^ or other state and municipal programs that can also be activated ^( 30 )^ .
*Right to Social Care*	SUAS ^†^ care services	Recognition of people with tuberculosis as meeting eligibility criterion for access to care services ^( 29 )^ .
		Ensure access to care services for people with tuberculosis who are homeless, assessing the possibility of stay for at least six months of treatment ^( 29 - 30 , 47 )^ .
		Creation of and access to Care Homes for people affected by tuberculosis, with social vulnerabilities, who lack family support for health care ^( 24 - 25 )^ .
*Right to Social Security*	Retirement due to permanent disability	Exemption from the grace period for formal or self-employed workers who contribute to the INSS ^‡‡^ for granting retirement due to permanent disability when the work disability is due to active tuberculosis ^( 19 , 30 - 32 , 35 , 43 )^ .
	Temporary disability benefit (sick pay)	Exemption from the grace period for formal or self-employed workers who contribute to the INSS ^‡‡^ for granting temporary disability benefit, that is, if they are unable to work for more than 15 consecutive days due to illness caused by active tuberculosis ^( 30 - 32 , 35 , 43 )^ .
	Exemption from income Tax	Exemption on income related to retirement, pension or military retirement, including supplementation received from a private entity and payment of living expenses for people affected by serious diseases, such as active tuberculosis ^( 27 , 29 - 30 , 34 , 46 )^ .
	Movement of money from PIS/PASEP ^§§^	Possibility of moving money from the PIS/PASEP ^§§^ account for holders or dependents with active tuberculosis ^( 21 )^ .
*Sharing of responsibilities*	Coordination between SUAS ^†^ and SUS*	Operation of SUAS ^†^ in coordination with SUS* to address tuberculosis through integration between the Social Care Network and the RAS ^||||(6,20,24,28-30,33,38-39,41,45,47)^ .
		Provision by SUAS ^†^ professionals of information on BCG vaccination ^¶¶^ , the importance of people with suspected tuberculosis seeking a close health care service, and support for the completion of treatment ^( 29 )^ .
		Establishment of mechanisms for coordination and sharing of care between the social care services of Basic Social Protection and Special Social Protection of medium and high complexity with the services of the RAS ^||||(28-29,47)^ .
	Intersectoral and interministerial actions	Coordination between the Ministry of Citizenship and the Ministry of Health to address tuberculosis, especially for people in situation of social vulnerability ^( 33 , 41 , 45 , 47 )^ .
		Interministerial coordination ^( 6 , 33 , 35 )^ and operation of CIEDDS*** within the scope of the Ministry of Health ^( 40 , 42 , 44 )^ .
		Intersectoral coordinations with strategic areas such as Primary Health Care, Indigenous Health Care, Mental Health Care, Occupational Health Care and multisectoral action through the Brazilian Network of Committees for Tuberculosis Control, the Brazilian Nursing Network to End Tuberculosis as a Public Health Problem, the Brazilian Partnership to Fight Tuberculosis and the Parliamentary Front to Fight Tuberculosis ^( 6 )^ .
	Speed of justice	Priority in the processing of judicial and administrative proceedings in which the person affected by tuberculosis is a party or interested party ^( 26 , 30 )^ .

*SUS = Unified Health System; ^†^SUAS = Unified Social Care System; ^‡^Pop Center = Specialized Referral Center for Homeless Population; ^§^FGTS = Guarantee Fund for Length of Service; ^||^CRAS = Referral Center for Social Care; ^¶^CREAS = Specialized Referral Center for Social Care; **BPC = Continuous Cash Benefit; ^††^PBF = Family Grant Program; ^‡‡^INSS = National Social Security Institute; ^§§^PIS/PASEP = Social Integration Program and Public Servant Asset Formation Program; ^||||^RAS = Health Care Network; ^¶¶^BCG = Bacillus of Calmette and Guérin; ***CIEDDS = Interministerial Committee for Elimination of Tuberculosis and Other Socially Determined Diseases

## Discussion

In the Brazilian context, considering the importance of people affected by TB being considered as full subjects, social protection policies have the goal of providing the population with access to their fundamental rights, in order to guarantee the exercise of citizenship, the meeting of basic needs and the promotion of human dignity^(31)^. In addition, they materialize the legal, ethical and moral importance in relation to the effectiveness of their response in TB treatment outcomes and relief of suffering among people and families affected by the disease^(48)^.

A study indicates that the availability and free distribution of antituberculosis drugs is not sufficient for the continuity of TB treatment or cure, since adherence to treatment is not reduced to an exclusively individual will, but is associated with other dimensions that are transversal to social production and reproduction processes^(49)^. This suggests a coordinated approach that includes not only health care services, but also an intra and intersectoral coordination based on a robust social protection system.

Accordingly, it is necessary to rethink public policies that represent own agendas and understand what they translate, their historical construction and the power relations established, which take into account the wishes and needs of the population. That is because the institutions of power are not really concerned with policies as a social instrument, other than those of a merely welfare nature that only attenuate social inequalities^(50)^.

After the promulgation of the 1988 Federal Constitution, which presents the Brazilian social security system, the intersection between health and human rights was revitalized with the inclusion of health as “*everyone’s right and the duty of the State*”^(15)^. Based on this context, we found the construction of a heterogeneous path of public policies for the social protection of people affected by TB in the country, marked by different political, social and ideological periods, with the resumption of visibility only 21 years after the promulgation of the Brazilian Constitution and the amendment of the legislation regarding the income tax statement^(27)^, with a law aimed at speeding up judicial and administrative proceedings, which included people with TB^(26)^.

Subsequently, 2019 was an important milestone for the discussion of TB as a public health problem that goes beyond the scope of health care. That year saw the publication of two normative documents — the Manual of Recommendations for TB Control in Brazil^(32)^ and Joint Operational Instruction No. 1/2019^(47)^ —, which support and guide most public policies aimed at social protection that were traced in this study, since they present strategies to strengthen intra and intersectoral coordination to guarantee human rights and citizenship in TB prevention and care actions^(32,47)^.

As a result, in order to align with Pillar 2 of the *End TB Strategy*, focused on bold policies and support systems, TB begins to be included more significantly in the political agendas of the Brazilian governmental spheres, through normative documents articulated with the executive and legislative branches, as well as in the work agendas of health care, social security and social care that enable social protection for people affected by TB^(32)^. From this perspective, TB prevention and care actions should be oriented toward addressing social inequalities and health inequalities that affect people with TB and their families, aiming at breaking cycles of poverty and misery, as well as sustainability in the creation of employment and income^(2)^.

It is estimated that TB morbidity and mortality can cause a serious impact on the global economy of about 1 trillion dollars between 2015 and 2030^(51)^. On the other hand, a study discusses that government-based social protection strategies, with a solid political commitment and adequate financial resources, have greater potential to achieve the global goal of 85% success in TB treatment^(52)^. To this end, one of the main issues refers to the guarantee of public investment, so both the centralized budget of the Ministry of Health and the decentralized resources through state or municipal funds are expanded by the three spheres of Brazilian management^(42)^.

This scenario is only possible through public policies that impact the production of health in the territory, such as those geared toward food and nutrition security, housing, sanitation, water, employment, income, education, transportation, among others^(40)^, revisiting the expanded concept of health recommended in the Federal Constitution and in the Organic Health Law^(17)^.

It was also possible to determine the leading role of SUS in the prevention and comprehensive care of people affected by TB, considering the transversality of the right to health. However, although the SUS offers universal and free treatment^(32)^, it is understood that TB causes losses and aggravates the socioeconomic situation, which can destabilize the family dynamics by creating additional expenses with food, transportation, other medicines and exams, for example^(39,47)^.

These direct or indirect expenses, when exceeding 20% of annual family income, are considered catastrophic costs generated by TB^(53)^ and directly impact the adherence and outcome of TB treatment, in addition to causing social sequelae^(54)^. To mitigate such costs or even — boldly — achieve the goal that no person with TB needs to bear catastrophic costs or social repercussions from the disease^(3)^, it is necessary to ensure that people with TB and affected families have access to social protection interventions against financial risks arising from treatment^(55)^.

One of the proposals for this situation is Bill (PL) No. 6,991, of 2013, whose objective is to guarantee financial support to families in poverty that have, among its members, people affected by TB, through the payment of a financial benefit in the amount of half a minimum wage for the duration of TB treatment^(56)^. This Bill, even 10 years after its drafting, is still being processed in the Chamber of Deputies, which may indicate a lack of priority for the approval of legislation that deals with conditional transfer of income to address diseases determined by poverty, such as TB.

Planning and implementing health care policies in cohesion with social policies can be highly effective in countries with a high TB burden. A successful example of this in the Region of the Americas is Argentina, which, although not on the list of 30 countries in this situation, implemented the conditional cash transfer policy for people affected by TB through Decree No. 170/91 of Law 10,436 of state funding for the payment of a minimum wage to all eligible people linked to the Provincial TB Control Program. This economic protection law was fundamental to motivate and monitor cases of difficult management, increasing adherence to TB treatment^(57-58)^.

It is important to emphasize that the rights of people affected by TB are the same as those of the population as a whole, without distinction, although there are situations and conditions for access to specific rights in the scope of health care, social care, and social security. In this sense, this study found a set of measures that covered public policies aimed at social inclusion, addressing poverty and ensuring access to social rights, food and nutrition security, transportation, work, housing and social security benefits for people affected by TB.

Some of these social protection strategies are more focused on the TB treatment period and are characterized as “TB-specific actions,” which benefit people affected by the disease and their families and are incorporated into existing TB treatment programs. Others are part of an expanded social security scheme, with great potential to modify structural conditions in society, by strengthening economic resilience, alleviating poverty and acting on other social determinants intrinsic to the illness and continuity of the TB transmission chain^(59)^.

A study carried out in Brazil found the potential in the implementation of specific actions for TB, such as the provision of food vouchers to people affected by TB, which increased the cure rate by 13% compared to the group without such intervention^(60)^. Regarding sensitive actions for TB, studies have presented one of the largest Brazilian examples: the Family Grant Program (PBF), which, through conditional income transfer, was responsible for a 7.6%^(61)^ and 8%^(62)^ higher cure rate and a 7% lower follow-up loss in groups of PBF beneficiaries with TB^(61)^.

However, it is important to understand the challenges that permeate the consolidation of these policies in the fight against TB. In order to guarantee the rights analyzed in this study, access to social protection policies involved conditions, such as the need for civil documentation, registration in the Single Registry for access to SUAS, or mandatory contribution to the INSS for access to social security benefits, which can imply barriers to the exercise of rights, especially for populations in situations of greater social vulnerability, such as homeless people.

That is because, in addition to this population being considered the most vulnerable to TB — with a 56 times higher risk when compared to the general population of the country^(30)^ —, there is evidence of the discrimination and invisibility of such people, due to lack of documents, home, stereotype or drug use, which reinforces the lack of care, deprivation and restriction of rights, citizenship and the very human condition^(63)^.

During the COVID-19 pandemic, there were indications that these issues intensified even more, considering the overlapping of social and programmatic vulnerabilities and the impact of restrictive measures on society and TB prevention and care^(64-65)^. In order to overcome barriers to access rights and comprehensive health care, some normative documents were prepared in this period aiming at the social protection of these people, with emphasis on the coordination and sharing of care between the Pop Center and Street Clinics teams^(37,39)^.

The regulations to address the stigmatization and discrimination of TB in the country follow a movement to combat the conceptions that marked the experience of TB in the past and that even today impose restrictions and obstacles to treatment, since they complicate the process of care and marginalize people affected by the disease^(30,47)^. Thus, documents that present measures for this type of combat enable recovering the right to situations that are not humiliating, degrading or that offend human dignity^(48)^.

Intersectoral operation, such as between SUS and SUAS, and interministerial coordination through the Interministerial Committee for Elimination of Tuberculosis and Other Socially Determined Diseases (CIEDDS)^(44)^ advance in the goal of prioritizing strategies that promote social protection. This prioritization can be effected through synergism between ministerial areas that are strategic to tackle the social determinants of TB in the areas of social care, justice and public security, work and income, human rights, racial equality, Indigenous peoples, education and citizenship^(40,42,44)^. Moreover, it is important to emphasize the importance of social responsibility and the involvement of areas such as nursing, which can substantially contribute to reduce underlying social and health inequalities in different community contexts^(66)^.

As limitations of this study, it should be noted the updating of the search platforms, which, despite having retrieved a robust volume of information, have weaknesses as to the availability of materials, and some normative documents may not have been found because they are not available electronically. Furthermore, the discussions raised have a critical-reflective content of the researchers and may not have covered other interpretations also applicable in the dispute arena of Brazilian public policies, with the analytical focus on social protection for people affected by TB.

Finally, the results of this documentary research advance scientific knowledge in the field of public health by expanding the understanding of the complex interactions between the right to health, social rights and social justice in the context of addressing TB, providing contributions to guide the formulation and implementation of more effective and inclusive public policies aimed at reducing social and health inequalities that strongly affect people with TB and their families.

## Conclusion

This study traced and analyzed normative documents that seek to guarantee the right to social protection through public policies oriented toward addressing TB that cover the right to health care, social care and social security, in addition to actions that involve the sharing of responsibilities for the effective exercise of such rights.

Such policies are considered recent in the history of the Democratic Rule of Law and there is still much room for improvement toward a comprehensive approach, focused on people affected by TB and that are, in fact, based on rights for prevention, care and support through intersectoral and interministerial coordination, with robust operation of SUS and SUAS.

However, the challenges posed by the difficult or lacking access to these rights, associated with other situations that increase social vulnerability, cause people affected by TB to remain socially unprotected. Accordingly, it is worth reaffirming the State’s role and duty to guarantee social protection by means of public policies that promote life, citizenship, human rights, and social justice.
