# Supplementary material for: Public social protection policies for people affected by tuberculosis: a documentary analysis
Source: Rev Lat Am Enfermagem. 2025 Mar 14;33:e4503. doi: 10.1590/1518-8345.7526.4503 (PMC11912822; doi:10.1590/1518-8345.7526.4503)
Supplement: Translation

# Translation: Políticas públicas para a proteção social às pessoas acometidas por tuberculose: uma análise documental*

**DOI:** 10.1590/1518-8345.7526.4504

**Authors:** Melisane Regina Lima Ferreira, Jaqueline Garcia de Almeida Ballestero, Rubia Laine de Paula Andrade, Tiemi Arakawa, Inês Fronteira, Aline Aparecida Monroe

**Affiliations:** 1 Universidade de São Paulo, Escola de Enfermagem de Ribeirão Preto, Centro Colaborador da OPAS/OMS para o Desenvolvimento da Pesquisa em Enfermagem, Ribeirão Preto, SP, Brasil; 2 Bolsista da Coordenação de Aperfeiçoamento de Pessoal de Nível Superior (CAPES), Brasil; 3 Ministério da Saúde, Coordenação Geral de Vigilância da Tuberculose, Micoses endêmicas e Micobactérias não tuberculosas, Departamento de HIV/Aids, Tuberculose, Hepatites Virais e Infecções Sexualmente Transmissíveis, Brasília, DF, Brasil; 4 Universidade NOVA de Lisboa, Escola Nacional de Saúde Pública, Lisboa, Portugal; 5 Bolsista do Conselho Nacional de Desenvolvimento Científico e Tecnológico (CNPq), Brasil

**Keywords:** Tuberculose, Política Pública, Seguridade Social, Política de Saúde, Direito Humanos, Previdência Social

## Abstract

analisar os documentos normativos que buscam garantir o direito à proteção social às pessoas acometidas por TB vigentes no Brasil em 2023.

pesquisa qualitativa, do tipo documental, realizada em setembro de 2023, a partir do levantamento de documentos de âmbito nacional, estadual e municipal, provenientes de órgãos governamentais e instâncias de controle social após a promulgação da Constituição Federal, em quatro plataformas eletrônicas, exportados e organizados no *software* Atlas.ti, e interpretados com base na análise de conteúdo, modalidade temática.

o *corpus* analítico foi composto por 30 documentos normativos, sendo nove leis, sete materiais técnicos-institucionais, cinco portarias, quatro resoluções, dois decretos, um acordo de cooperação técnica, uma instrução normativa e uma instrução operacional, dos quais emergiram quatro categorias temáticas: o direito à saúde, o direito à assistência social, o direito à previdência social e o compartilhamento de responsabilidades.

as políticas de proteção às pessoas com tuberculose no Brasil são recentes e ainda há um longo caminho para uma abordagem abrangente por meio de articulações intersetoriais e interministeriais, visando ao enfrentamento da vulnerabilidade social e reafirmando o dever do Estado em garantir proteção social por meio de políticas públicas que promovam vida, cidadania, direitos humanos e justiça social.

## Destaques

**(1)** O direito à saúde inclui políticas públicas dentro do SUS para o enfrentamento da TB. **(2)** O direito à assistência social destacou o acesso a serviços, programas e benefícios. **(3)** O direito à previdência social abrangeu aposentadorias e auxílios para pessoas com TB. **(4)** Responsabilidade interministerial, intersetorial e compartilhada entre SUS e SUAS. **(5)** Desafios persistem no acesso aos direitos sociais e de saúde pelas pessoas com TB.

## Introdução

A tuberculose (TB) é uma das doenças mais emblemáticas relacionadas à pobreza e fortemente atravessada pelos determinantes sociais. Paralelamente, é considerada perpetuadora de ciclos de miserabilidade devido aos impactos sociais e econômicos gerados pelo adoecimento^(1)^. Como doença multicausal, a TB exige uma resposta multissetorial e um sinergismo entre políticas públicas de proteção social que promovam a eliminação da pobreza, a equidade, a justiça e os direitos humanos das pessoas acometidas pela doença, incluindo o combate a toda forma de discriminação e estigma^(2)^.

A estratégia *The End TB,* da Organização Mundial da Saúde (OMS), recomenda a redução de 90% das taxas incidência e 95% das taxas de mortalidade até 2035 para eliminá-la como endemia até 2050, tendo como meta adicional que nenhuma pessoa com TB precise arcar com custos catastróficos ou repercussões sociais pela doença^(3)^. Em consonância, por meio dos Objetivos do Desenvolvimento Sustentável (ODS) das Nações Unidas, a TB está incluída na Meta 3.3 do terceiro ODS, na qual há a propositura de reduzir 90% das mortes por TB e 80% dos casos novos da doença até 2030. Tal meta também se relaciona intimamente com o décimo objetivo referente à redução das desigualdades^(4)^.

No entanto, em 2022, estimou-se que a TB tenha acometido 10,6 milhões de pessoas no mundo, com 1,3 milhão de óbitos pela doença e outras 167 mil mortes entre pessoas vivendo com HIV/aids. No mesmo ano, a região das Américas foi responsável por 3,1% do total de casos de TB mundialmente e um número estimado de mortes causadas pela doença muito maior do que em 2015 (+41%)^(3)^. Nesta região, apenas o Brasil está incluído na lista dos 30 países com as maiores cargas de TB mundialmente, visto que, em 2022, o país foi responsável por 78.057 casos novos de TB, com um coeficiente de incidência de 36,3 casos/100 mil habitantes, além de 5.162 óbitos pela doença, com um coeficiente de mortalidade de 2,2 óbitos/100 mil habitantes^(5)^.

Isso implica dizer que o país ainda se encontra distante do alcance das proposituras da estratégia *The End TB* e do Plano Nacional pelo Fim da TB como problema de saúde pública, que preconizam até 2035 a redução do coeficiente de incidência da doença para menos de 10 casos/100 mil habitantes e um óbito/100 mil habitantes^(6-7)^. Ao considerar este cenário, uma das propostas mais ousadas para o fim da TB se sustenta no 2º Pilar referente às políticas arrojadas e aos sistemas de apoio, almejando a proteção social das pessoas acometidas pela doença, além da diminuição da pobreza e outros fatores determinantes para o adoecimento por TB^(3)^.

A proteção social diz respeito às ações que envolvem políticas públicas que visam garantir a vida e os direitos humanos, prevenir a incidência de riscos e vulnerabilidades, reduzir danos e repercussões negativas por decorrência de restrições sociais, econômicas, políticas, naturais ou de ofensas à dignidade humana^(8)^. Para o enfrentamento da TB, revisão de literatura realizada em uma perspectiva global evidenciou que medidas e estratégias destinadas à proteção social enquanto um direito das pessoas acometidas por TB melhoram o estado nutricional, a qualidade de vida e a adesão ao tratamento, reduzindo custos catastróficos e promovendo resultados favoráveis^(9)^.

Além disso, um estudo que analisou globalmente a associação entre gastos com proteção social e carga de TB evidenciou que países que investem uma elevada parte de seu Produto Interno Bruto (PIB) em políticas de proteção social vivenciam menores taxas de prevalência, incidência e mortalidade por TB^(10)^. Tais questões evidenciam o alinhamento da proteção social às políticas públicas propostas para a superação do desafio de eliminar a TB como problema de saúde pública em nível nacional e internacional.

Entretanto, ainda existem lacunas na compreensão de como as medidas e estratégias de proteção social estão regulamentadas enquanto políticas públicas, bem como estão orientadas para implementação em diferentes contextos socioeconômicos. A partir disso, este estudo pretende contribuir para o avanço do conhecimento científico ao explorar como as políticas protetivas às pessoas com TB estão normatizadas em âmbito nacional e como podem ser adaptadas de forma mais inclusiva para melhorar os mecanismos de suporte e tratamento disponíveis, impactando a qualidade de vida das pessoas acometidas pela doença e a eficiência dos sistemas de saúde.

Neste sentido, políticas públicas destinadas à superação das desigualdades que repercutem na saúde humana e por si só se caracterizam como injustas são fundamentais para a conquista de uma sociedade mais equitativa e que acarretem, portanto, impacto no controle da TB^(11)^. Logo, é oportuna uma análise de conjuntura considerando as potencialidades do Brasil e os futuros desafios que a crise financeira e as políticas de austeridade possam fomentar no sistema de saúde e socioassistencial brasileiros.

Assim, guiados pela pergunta de pesquisa “como a proteção social às pessoas acometidas pela TB se insere no arcabouço das políticas públicas brasileiras?”, objetivou-se analisar os documentos normativos que buscam garantir o direito à proteção social às pessoas acometidas por TB vigentes no Brasil em 2023.

## Método

### Delineamento do estudo

Trata-se de uma pesquisa qualitativa do tipo documental, realizada a partir de documentos que regulamentam as políticas públicas que incluem em seu escopo medidas de proteção social às pessoas acometidas por TB no cenário nacional brasileiro. Esse tipo de pesquisa é caracterizado pela busca de informações em documentos com dados originais a partir de métodos e técnicas para a apreensão, compreensão e análise de informações factuais pelo pesquisador, uma vez que os documentos não passaram antes por nenhum tratamento científico^(12-13)^.

A utilização de documentos em uma pesquisa permite o resgate de uma riqueza de informações que possibilitam ampliar o entendimento de temáticas cuja compreensão necessita de uma contextualização histórica e sociocultural^(12)^. Nesse sentido, os documentos oficiais são considerados fontes fidedignas de dados, por possibilitarem uma análise contextual dos atos normativos e políticos^(14)^.

### Critérios de seleção

Optou-se pela inclusão de documentos posteriores ao marco histórico-temporal da Constituição Federal de 1988, que garante a proteção social no Brasil por meio do tripé formado pelas políticas de saúde, de assistência social e da previdência social, as quais constituem a seguridade social no país^(15)^. A partir desse contexto, houve o levantamento de materiais técnicos institucionais, bem como de legislações, decretos, portarias, resoluções e outros atos normativos nacionais, estaduais ou municipais, provenientes dos órgãos governamentais e de suas instâncias de controle social, que estejam vigentes.

Foram excluídos documentos relacionados à incorporação de novos regimes terapêuticos antituberculose, testes diagnósticos para a TB, consultas públicas, projetos de lei, documentos instituídos por órgãos internacionais, bem como normativas que não especificassem medidas de proteção social direcionadas às pessoas acometidas por TB.

### Levantamento documental

As buscas foram realizadas pela pesquisadora principal, em setembro de 2023, nas seguintes plataformas eletrônicas: Diário Oficial da União (DOU) (https://www.in.gov.br/consulta/) para documentos federais, estaduais e/ou municipais; Portal da Legislação Federal Brasileira (https://legislacao.presidencia.gov.br/) para documentos federais; Portal das Leis Estaduais (https://leisestaduais.com.br/) para documentos estaduais e/ou municipais; e site oficial de publicações do Ministério da Saúde (https://www.gov.br/saude/pt-br/centrais-de-conteudo/publicacoes) para documentos federais, a partir da palavras-chave: “Tuberculose” e “Proteção Social”.

Para as buscas no DOU, visando otimizar o processo de levantamento documental, foi utilizado o filtro “Organização Principal” para selecionar documentos emitidos por órgãos com competência direta sobre as políticas de proteção social e “Tipo de Ato” para selecionar atos específicos que estabelecem ou regulamentam políticas e práticas relacionadas à proteção social, com relevância normativa e legal. Além disso, para as consultas de documentos com datas anteriores a 01/01/2018, foram utilizados os filtros “Pesquisa na Versão Certificada” ou “Diário Completo Certificado”. Para o site do Ministério da Saúde, foi utilizado o filtro “todas as publicações”, e para as demais plataformas foram utilizados os filtros de “normas em vigor” ou “publicadas” e recorte temporal a partir de 1988.

Cabe ressaltar que, visando reduzir um possível viés de interesse na utilização do Portal das Leis Estaduais, foram adotadas medidas quanto à transparência na escolha das legislações estaduais e municipais, bem como uma análise rigorosa dos dados, para evitar distorções e preservar a integridade e a imparcialidade do presente estudo. Destaca-se também que não foram incluídos documentos provenientes de *sites* da sociedade civil organizada, uma vez que o objetivo do estudo estava relacionado a examinar as políticas implementadas e não as demandas dos diferentes atores sociais.

### Tratamento e análise dos dados

Posteriormente ao levantamento e acesso às publicações oficiais, os documentos foram salvos em formato PDF e exportados para o *software* Atlas.ti, versão 23, no intuito de organizá-los e interpretá-los, utilizando a análise de conteúdo modalidade temática, a qual foi desenvolvida em três etapas segundo os pressupostos de Bardin^(16)^, a saber: pré-análise documental; exploração do material; e tratamento dos resultados. Na etapa de pré-análise, conduzida integralmente por duas pesquisadoras, incluindo a principal, realizou-se a leitura flutuante dos textos, a fim de identificar sua relação com o objetivo do estudo e a formulação de núcleos de sentido a partir de cinco dimensões^(14)^: análise do contexto; da autoria; interesses e/ou confiabilidade do texto; natureza do texto; e conceitos-chave do texto.

Em seguida, procedeu-se à exploração do material por meio da leitura em profundidade dos documentos que constituíram o *corpus* analítico. Para a extração das informações de tais documentos realizada pela pesquisadora principal, foi utilizada uma planilha do *Microsoft Excel* para codificá-los e categorizá-los quanto a tipologia, ano de publicação, órgão ou instituição de origem, abrangência e tópicos e/ou temas tratados. Tais categorias foram certificadas por outras duas pesquisadoras, cuja etapa de certificação permeou a revisão e validação das categorias em relação aos dados extraídos e à conformidade com o objeto do estudo.

Na etapa de tratamento dos resultados, foi realizada estatística descritiva simples dos dados relativos à categorização dos documentos. Por fim, os achados foram interpretados pelas três pesquisadoras diretamente envolvidas nas etapas anteriores (incluindo a principal), de acordo com o questionamento do estudo, para serem analisados na perspectiva do direito à proteção social, que abrange medidas voltadas à garantia da saúde, assistência e previdência social. Destaca-se que este estudo se fundamenta em uma concepção ampliada de saúde proposta pelo movimento da Reforma Sanitária brasileira e na noção de direito como conquista social, devendo o Estado prover as condições indispensáveis ao seu pleno exercício e assegurar acesso universal e igualitário às ações e aos serviços para a sua promoção, proteção e recuperação^(17)^.

### Aspectos éticos

Embora não seja necessária a aprovação ética para a realização de uma pesquisa documental, pontua-se que este estudo compõe uma pesquisa sobre a avaliação da proteção social às pessoas acometidas por TB, aprovada pelo Comitê de Ética em Pesquisa da instituição responsável, sob o parecer número 6.389.278, de 05/10/2023 (CAAE 71246023.6.0000.5393). Além disso, procurou-se utilizar uma linguagem não estigmatizante relacionada à TB durante todo o estudo^(18)^.

## Resultados

O levantamento documental junto às plataformas eletrônicas selecionadas neste estudo resultou na inclusão de 30 documentos normativos publicados por meio de nove leis (30%)^(19-27)^, sete materiais técnicos-institucionais (23,4%)^(6,28-33)^, cinco portarias (16,7%)^(34-38)^, quatro resoluções (13,3%)^(39-42)^, dois decretos (6,7%)^(43-44)^, um acordo de cooperação técnica (3,3%)^(45)^, uma instrução normativa (3,3%)^(46)^ e uma instrução operacional (3,3%)^(47)^, no período entre 1988 e agosto de 2023, sendo o ano de 2022 aquele com o maior número de publicações (n= 08, 26,6%) (Figura 1).

**Figura 1 -:** Linha histórica representativa dos caminhos das políticas públicas para a proteção social às pessoas acometidas por TB no Brasil

*PRES/INSS = Perícia Médica do Instituto Nacional do Seguro Social; ^†^DIRBEN/INSS = Diretoria de Benefícios do Instituto Nacional do Seguro Social; ^‡^MTP = Ministério do Trabalho e Previdência; ^§^MS = Ministério da Saúde

As normativas incluídas que regulamentam ou orientam a proteção social às pessoas acometidas por TB (Figura 2) foram provenientes majoritariamente de órgãos governamentais de abrangência nacional (n=22, 73,3%), tais como a Casa Civil^(21-22,26-27,43-44)^, o Ministério dos Direitos Humanos e da Cidadania^(36-39,45,47)^, o Ministério da Saúde^(28-33)^ e o Ministério do Trabalho e Emprego^(34-35,46)^; estaduais (n= 05, 16,7%), como a Assembleia Legislativa do Mato Grosso^(20)^ e governos estaduais do Rio de Janeiro^(23,25,41)^ e Rio Grande do Norte^(19)^; e de abrangência municipal (n=01, 3,3%), por meio da Câmara Municipal do Rio de Janeiro^(24)^. Além disso, duas publicações (6,7%) foram oriundas do Conselho Nacional de Saúde^(40,42)^.

**Figura 2 -:** Caracterização dos documentos normativos que buscam garantir o direito à proteção social às pessoas acometidas por TB, quanto ao seu nome, definições e articulação com a proteção social às pessoas acometidas por TB (n = 30)

**Nome do documento**	**Definições e articulação com a proteção social às pessoas acometidas por TB**
Acordo de Cooperação Técnica nº 60/2021 ^( 45 )^	- Integração de políticas públicas de saúde à assistência social;
	- Benefícios sociais às pessoas em situação de vulnerabilidade social e mais suscetíveis à tuberculose;
	- Desenvolvimento de programas específicos que abordem as necessidades das pessoas com tuberculose.
Decreto nº 10.410, de 30 de junho de 2020 ^( 43 )^	- Ajusta as regras de acesso a benefícios previdenciários, como auxílio-doença, aposentadoria por invalidez e outros benefícios que são essenciais para trabalhadores incapacitados temporária ou permanentemente devido à tuberculose.
	- Contribui para a prevenção da vulnerabilidade socioeconômica das pessoas acometidas por tuberculose.
Decreto nº 11.494, de 17 de abril de 2023 ^( 44 )^	- Estabelecimento de um comitê que coordene esforços interministeriais para enfrentar os determinantes sociais da tuberculose;
	- Integração e implementação de políticas que protejam os direitos e o bem-estar das pessoas com tuberculose.
Instrução normativa PRES/INSS* nº 128, de 28 de março de 2022 ^( 46 )^	- Estabelece procedimentos claros e acessíveis para o acesso a benefícios previdenciários, assegurando que as pessoas com tuberculose recebam o apoio financeiro necessário durante o tratamento e a recuperação.
	- Oferece uma rede de segurança financeira para as pessoas com tuberculose e ajuda a mitigar o impacto econômico da doença.
Instrução Operacional Conjunta nº 1, de 26 de setembro de 2019 ^( 47 )^	- Promove uma colaboração entre saúde e assistência social necessária para superar as barreiras impostas pela tuberculose, como perda de renda, necessidade de apoio alimentar, outras vulnerabilidades e exclusão social.
Lei Complementar nº 570, de 26 de abril de 2016 ^( 19 )^	- Reforça a proteção social das pessoas com tuberculose ao garantir que elas tenham acesso a uma aposentadoria por invalidez com proventos integrais, proporcionando-lhes segurança financeira e reconhecendo a gravidade da doença em termos legais e previdenciários.
Lei nº 11.576, de 17 de novembro de 2021 ^( 20 )^	- Cria uma política estadual específica para o controle e a eliminação da tuberculose por meio da articulação com outras áreas, como assistência social, habitação e alimentação, que são fundamentais para a proteção social das pessoas acometidas.
Lei nº 13.677, de 13 de junho de 2018 ^( 21 )^	- Proporciona acesso a recursos financeiros que podem ser usados para suprir necessidades emergentes durante o tratamento da tuberculose e alívio financeiro, complementando outras formas de suporte social e econômico.
Lei nº 14.289, de 3 de janeiro de 2022 ^( 22 )^	- Promove a proteção social ao prevenir o estigma e a discriminação relacionados à tuberculose ao garantir direitos humanos e dignidade e criar um ambiente mais seguro e confidencial, para que as pessoas possam buscar e continuar o tratamento sem medo de exposição indevida.
Lei nº 6.541, de 19 de setembro de 2013 ^( 23 )^	- Contribui para a proteção social ao facilitar o acesso ao tratamento, reduzir os custos associados à doença, incentivar a continuidade do tratamento e promover a inclusão social das pessoas com tuberculose.
Lei nº 7.286, de 31 de março de 2022 ^( 24 )^	- Cria uma política municipal específica para o controle e a eliminação da tuberculose por meio da articulação com outras áreas, como assistência social, habitação e segurança alimentar, que são fundamentais para a proteção social das pessoas acometidas.
Lei nº 8.746, de 09 de março de 2020 ^( 25 )^	- Cria uma política estadual específica para o controle e a eliminação da tuberculose por meio de garantia aos serviços de saúde, coordenação de políticas públicas e proteção contra discriminação e exclusão social.
Lei nº 12.008, de 29 de julho de 2009 ^( 26 )^	- Contribui para a proteção social das pessoas com tuberculose ao facilitar o acesso rápido e eficiente a direitos e benefícios, reduzir burocracia e atrasos e fornecer uma proteção adicional e suporte jurídico adequado para aqueles que enfrentam a doença.
Lei nº 7.713, de 22 de dezembro de 1988 ^( 27 )^	- Ao permitir isenções e deduções fiscais para pessoas acometidas por tuberculose, pode reduzir a carga financeira, aliviar os custos associados ao tratamento e promover um suporte econômico crucial para o manejo da doença.
Assistência do enfermeiro à pessoa com tuberculose na Atenção Primária ^( 28 )^	- Vinculado à proteção social ao assegurar acesso a cuidados adequados, promover a educação, realizar monitoramento contínuo, identificar e encaminhar as pessoas com tuberculose para tratamento especializado, oferecer suporte psicossocial e coordenar com outros serviços.
Diretrizes Nacionais para articulação entre o Sistema Único de Saúde e o Sistema Único de Assistência Social para a promoção do cuidado integral e da proteção social das pessoas em situação de vulnerabilidade e risco social, vivendo com HIV/aids, Sífilis, Hepatites Virais, Hanseníase e Tuberculose ^( 29 )^	- Estabelece uma estrutura para a integração de cuidados de saúde e assistência social;
	- Promove um cuidado integral e assegura que as pessoas com tuberculose recebam o suporte necessário para enfrentar suas necessidades complexas e melhorar sua qualidade de vida.
	- Enfrentamento das vulnerabilidades adicionais, como dificuldades financeiras, estigma e falta de acesso a serviços.
Guia orientador: promoção da proteção social para as pessoas acometidas pela tuberculose ^( 30 )^	- Orienta diretrizes para que as pessoas com tuberculose recebam um suporte abrangente e eficaz, abordando suas necessidades de saúde e sociais e promovendo um sistema de proteção social integrado;
	- Oferece informações sobre programas e políticas que visam reduzir a carga financeira sobre as pessoas com tuberculose e seus familiares.
Linha de cuidado da tuberculose: orientações para gestores e profissionais de saúde ^( 31 )^	- Fornece diretrizes para uma atenção coordenada, acessível e de alta qualidade, além de oferecer suporte adicional que melhora a adesão ao tratamento e a qualidade de vida das pessoas com tuberculose.
Manual de Recomendações para o controle da tuberculose no Brasil ^( 32 )^	- Estabelece diretrizes relacionadas à proteção social por meio do cuidado eficaz e integrado entre saúde e assistência social;
	- Orienta a educação e o treinamento dos profissionais de saúde e assegura que as pessoas com tuberculose tenham acesso a recursos e suporte necessários para enfrentar a doença e suas consequências.
Plano Nacional pelo Fim da Tuberculose como problema de saúde pública: estratégias para 2021-2025 ^( 6 )^	- Estabelece uma abordagem estratégica e integrada entre políticas de saúde e políticas de proteção social para o controle da doença;
	- Recomenda ações para o acesso equitativo ao tratamento, reduzindo custos financeiros e melhorando o suporte social e os recursos disponíveis para as pessoas com tuberculose.
Rede brasileira de comitês para o controle da tuberculose no Brasil: passado, presente e futuro ^( 33 )^	- Aborda a coordenação de esforços entre diferentes entidades e níveis de governo para o desenvolvimento de políticas e práticas de proteção social;
	- Comitês bem estruturados e operacionais ajudam a assegurar que as pessoas com tuberculose tenham acesso a cuidados, suporte social e recursos necessários para enfrentar a doença.
Portaria DIRBEN/INSS ^†^ nº 992, de 28 de março de 2022 ^( 34 )^	- Normatiza o acesso a benefícios previdenciários, como aposentadoria por invalidez ou auxílio-doença;
	- Garante que as pessoas com tuberculose recebam o suporte financeiro necessário para compensar a perda de renda devido à incapacidade para o trabalho.
Portaria interministerial MTP ^‡^ /MS ^§^ nº 22, de 31 de agosto de 2022 ^( 35 )^	- Define diretrizes para a colaboração entre os ministérios responsáveis pela saúde e pelo trabalho, assegurando uma abordagem coordenada que aborde as necessidades de saúde, as questões de emprego e a proteção social de forma integrada e eficaz das pessoas com tuberculose.
Portaria nº 100, de 14 de julho de 2020 ^( 36 )^	- Garante que os serviços socioassistenciais essenciais continuem a ser prestados durante emergências de saúde pública, assegurando a continuidade do cuidado e a adaptação dos serviços às novas condições, promovendo suporte tanto para o manejo da tuberculose quanto para a proteção social integral.
Portaria nº 54, de 1º de abril de 2020 ^( 37 )^	- Garante a continuidade dos serviços de assistência social durante contextos de crise em saúde pública, com foco em medidas de segurança e saúde para usuários e profissionais, assegurando que as pessoas com tuberculose recebam serviços críticos para a proteção social, como assistência financeira e apoio social, sem comprometer sua saúde.
Portaria nº 69, de 14 de maio de 2020 ^( 38 )^	- Aborda a circunstância da população em situação de rua durante emergências de saúde pública, garantindo que essas pessoas, que frequentemente enfrentam condições de vulnerabilidade adicionais, sejam protegidas socialmente e recebam cuidados necessários para o manejo de suas condições de saúde, incluindo a tuberculose.
Resolução nº 40, de 13 de outubro de 2020 ^( 39 )^	- Assegura que os direitos humanos e as necessidades específicas da população em situação de rua acometida por tuberculose sejam atendidos de forma integrada e abrangente por meio de políticas públicas alinhadas para protegê-las.
Resolução nº 719, de 17 de agosto de 2023 ^( 40 )^	- Define diretrizes e propostas que podem impactar diretamente as políticas de saúde e a assistência social para melhorar a prevenção, o tratamento e o suporte para pessoas com tuberculose.
Resolução nº 2.580, de 23 de dezembro de 2021 ^( 41 )^	- Garante a alocação de recursos para ações específicas que visam melhorar o cuidado e suporte às pessoas com tuberculose;
	- Fomenta a colaboração entre saúde e assistência social e reforça a capacidade dos municípios, promovendo uma proteção social mais robusta para a população afetada.
Resolução nº 709, de 16 de março de 2023 ^( 42 )^	- Contribui para um sistema de proteção social mais eficaz e holístico, garantindo que as pessoas com tuberculose recebam o suporte necessário para superar a doença e melhorar sua qualidade de vida, por meio da integração de políticas de saúde e assistência social.

*PRES/INSS = Perícia Médica do Instituto Nacional do Seguro Social; ^†^DIRBEN/INSS = Diretoria de Benefícios do Instituto Nacional do Seguro Social; ^‡^MTP = Ministério do Trabalho e Previdência; ^§^MS = Ministério da Saúde

Da análise documental emergiram quatro categorias temáticas que abrangeram suas respectivas unidades de sentido e os principais resultados identificados nos documentos normativos incluídos que compuseram o *corpus* do estudo (Figura 3). A primeira categoria temática, o *direito à saúde*, refere-se a documentos que apresentaram políticas sociais e econômicas para a garantia das ações de prevenção e cuidado da TB no Sistema Único de Saúde (SUS), da segurança alimentar e nutricional, transporte, trabalho, controle social e enfrentamento do estigma e discriminação da TB. Ressalta-se que nessa categoria também foram identificados documentos voltados especificamente à proteção social das pessoas em situação de rua acometidas por TB.

A segunda categoria temática, o *direito à assistência social*, incluiu documentos que apresentaram o acesso aos serviços, programas, projetos e benefícios socioassistenciais, o direito ao Benefício de Prestação Continuada (BPC), aos benefícios eventuais, à documentação civil, aos programas de transferência de renda e aos serviços de acolhimento do Sistema Único de Assistência Social (SUAS). O *direito à previdência social* constituiu a terceira categoria temática que abrangeu documentos que apresentaram o direito das pessoas acometidas por TB à aposentadoria por incapacidade permanente, ao auxílio por incapacidade temporária (auxílio-doença), à isenção de imposto de renda e à movimentação financeira vinculada ao Programa de Integração Social e ao Programa de Formação do Patrimônio do Servidor Público (PIS/PASEP).

A última categoria temática compreendeu documentos direcionados ao *compartilhamento de responsabilidades* no enfrentamento da TB, por meio da atuação do SUAS em articulação com o SUS, de ações intersetoriais e interministeriais, bem como normativas voltadas à celeridade da justiça para as pessoas acometidas pela TB.

**Figura 3 -:** Descrição da análise documental quanto às quatro categorias temáticas, unidades de sentido e principais resultados encontrados nos documentos normativos que buscam garantir o direito à proteção social às pessoas acometidas por tuberculose (n = 30)

**Categorias temáticas**	**Unidades de sentido**	**Principais resultados (documentos normativos)** |	
*Direito à Saúde*	Ações de prevenção e cuidado da tuberculose no SUS*	Atenção integral às necessidades de saúde, econômicas, psicológicas e sociais das pessoas acometidas com tuberculose ^( 20 , 24 - 25 )^ , através da realização de encaminhamentos às outras políticas públicas ^( 47 )^ .	
		Consolidação e fortalecimento da linha de cuidado da tuberculose, a fim de garantir o acesso ao cuidado e sua continuidade entre diferentes serviços e níveis de atenção ^( 42 )^ .	
		Identificação oportuna de pessoas com sinais e sintomas de tuberculose pelas equipes de saúde e assistência social ^( 30 )^ .	
		Fortalecimento e ampliação das ações de promoção à saúde, pelas equipes e programas da Atenção Básica, com ênfase na Estratégia Saúde da Família ^( 39 )^ .	
		Articulação do Projeto Terapêutico Singular do SUS* e do Plano Individual de Atendimento do SUAS ^†^ para atenção integral da pessoa com tuberculose e sua família ^( 47 )^ .	
		*População em situação de rua acometida por tuberculose*: articulação e compartilhamento do cuidado entre as equipes do Centro Pop ^‡^ com as equipes de saúde dos Consultórios na Rua ^( 37 , 39 )^ .	
	Segurança alimentar e nutricional	Garantia de alimentação saudável e adequada ^( 24 - 25 , 29 , 40 )^ por meio de incentivos como lanches ^( 28 )^ , leite ^( 28 )^ , recebimento de cestas básicas ^( 25 , 28 - 29 , 33 , 41 )^ , vale-alimentação ou cartão alimentação ^( 41 )^ .	
		Acesso regular e permanente de alimentos de qualidade e quantidade suficiente, por meio da utilização de restaurantes populares ^( 25 , 29 - 30 , 32 , 41 )^ , banco de alimentos, cozinhas comunitárias, feiras e mercados populares ^( 30 )^ e da integração com o Sistema Nacional de Segurança Alimentar e Nutricional ^( 47 )^ .	
	Transporte	Isenção de pagamento de tarifas nos serviços de transporte intermunicipal ^( 23 , 25 , 30 , 32 )^ , por meio da concessão de, no máximo, 60 vales sociais mensalmente em até 30 dias úteis ^( 23 )^ ou em até 15 dias úteis ^( 25 )^ .	
		Garantia do vale social ao acompanhante da pessoa com tuberculose que não possa circular desacompanhada ^( 25 , 32 )^ ou àquela incapaz de se locomover sozinha ^( 32 )^ .	
		Acesso ao transporte gratuito ^( 29 )^ por meio da disponibilização de vale-transporte ^( 28 )^ em barcas, metrôs, ônibus e trens ^( 32 )^ .	
	Trabalho	Programas de promoção do acesso ao mundo do trabalho ^( 29 )^ .	
		Manutenção das relações de trabalho por meio da proibição de demissão do trabalho por ter contraído tuberculose, garantia de ambientes salubres e saque do FGTS ^§(30)^ .	
	Controle social	Fomento à participação da representação da sociedade civil, de representantes e lideranças comunitárias e das pessoas acometidas por tuberculose nas instâncias de controle social do SUS* e SUAS ^†(29-30,33,45)^ .	
		Estabelecimento de fóruns de articulação entre assistência social e saúde ^( 45 )^ .	
	Combate ao estigma e à discriminação da tuberculose	Promoção de ações de inclusão social das pessoas com tuberculose ^( 30 , 45 )^ , dentro das unidades de saúde e socioassistenciais, no território e nos espaços comunitários ^( 6 , 47 )^ .	
		Proibição de submissão a tratamento desumano ou degradante, privação de liberdade ou do convívio familiar e discriminação por motivo de sua morbidade ^( 20 , 25 )^ .	
		Obrigatoriedade de preservação do sigilo sobre a condição da pessoa com tuberculose ^( 22 )^ .	
		Orientação sobre situações discriminatórias, sobre a utilização dos canais de comunicação e registro de denúncia por meio das ouvidorias ou serviços como o “Disque 100”, “Disque 180” e “Disque Saúde 136” ^( 29 )^ .	
*Direito à Assistência Social*	Acesso aos serviços, programas, projetos e benefícios socioassistenciais	Garantia do acesso e inclusão no Cadastro Único (CadÚnico) ^( 28 , 30 , 32 , 47 )^ para a oferta qualificada dos serviços, programas, projetos e benefícios socioassistenciais do governo federal às pessoas com tuberculose em situação de risco e vulnerabilidade ^( 6 , 28 , 31 , 45 , 47 )^ .	
		Reconhecimento das pessoas com tuberculose como critério de elegibilidade para os programas e serviços de assistência social ^( 29 , 32 )^ .	
		Utilização do CRAS ^||^ , CREAS ^¶^ , Centro Pop ^‡^ , Unidades de Acolhimento, entre outros, como espaços para o desenvolvimento de atividades visando à proteção social das pessoas acometidas por tuberculose ^( 28 - 29 , 47 )^ .	
	BPC**	Recebimento de um salário mínimo mensal para pessoas com deficiência de qualquer idade ou idosos, com idade de 65 anos ou mais, que não têm condições de se sustentar ou serem sustentados pela família ^( 30 , 32 )^ .	
	Benefícios eventuais	Acesso a benefícios financeiros, de caráter suplementar e temporário, em situações de nascimento, morte, vulnerabilidade provisória e de calamidade pública ^( 30 , 32 )^ .	
*Direito à Assistência Social*	Documentação civil	Acesso à documentação civil básica ^( 29 - 30 , 47 )^ .	
	Programas de transferência de renda	Reconhecimento das pessoas com tuberculose como critério de elegibilidade para os programas de transferência de renda ^( 29 )^ , como o PBF ^††^ ou outros programas estaduais e municipais que também podem ser acionados ^( 30 )^ .	
	Serviços de acolhimento do SUAS ^†^	Reconhecimento das pessoas com tuberculose como critério de elegibilidade para o acesso aos serviços de acolhimento ^( 29 )^ .	
		Garantir acesso aos serviços de acolhimento às pessoas com tuberculose que não tenham moradia, avaliando a possibilidade de permanência por pelo menos seis meses de tratamento ^( 29 - 30 , 47 )^ .	
		Criação e acesso a Casas de Acolhimento para pessoas acometidas por tuberculose, com vulnerabilidades sociais, que não tenham suporte familiar para os cuidados em saúde ^( 24 - 25 )^ .	
*Direito à Previdência Social Compartilhamento de responsabilidades*	Aposentadoria por incapacidade permanente	Isenção de período de carência aos trabalhadores com carteira assinada ou autônomos que contribuem com o INSS ^‡‡^ para concessão de aposentadoria por incapacidade permanente quando a incapacidade laborativa for a tuberculose ativa ^( 19 , 30 - 32 , 35 , 43 )^ .	
	Auxílio por incapacidade temporária (auxílio-doença)	Isenção de período de carência aos trabalhadores com carteira assinada ou autônomos que contribuem com o INSS ^‡‡^ para concessão de auxílio por incapacidade temporária, ou seja, caso fiquem incapazes para o trabalho por mais de 15 dias consecutivos em razão do adoecimento por tuberculose ativa ^( 30 - 32 , 35 , 43 )^ .	
	Isenção de imposto de renda	Isenção sobre os rendimentos relativos à aposentadoria, à pensão ou à reforma, incluindo a complementação recebida de entidade privada e a pensão alimentícia para pessoas acometidas por doenças graves, como a tuberculose ativa ^( 27 , 29 - 30 , 34 , 46 )^ .	
	Movimentação financeira do PIS/PASEP ^§§^	Possibilidade de movimentação da conta do PIS/PASEP ^§§^ pelos titulares ou dependentes com tuberculose ativa ^( 21 )^ .	
	Articulação SUAS ^†^ e SUS*	Atuação do SUAS ^†^ em articulação com o SUS* para o enfrentamento da tuberculose, por meio da integração entre a Rede Socioassistencial e a RAS ^||||(6,20,24,28-30,33,38-39,41,45,47)^ .	
		Orientação pelos profissionais do SUAS ^†^ quanto à vacinação da BCG ^¶¶^ , à importância de pessoas com suspeita de tuberculose procurarem um serviço de saúde mais próximo e ao apoio à conclusão do tratamento ^( 29 )^ .	
		Estabelecimento de mecanismos de coordenação e compartilhamento do cuidado entre os serviços socioassistenciais de Proteção Social Básica e de Proteção Social Especial de média e alta complexidade com os serviços da RAS ^||||(28-29,47)^ .	
	Ações intersetoriais e interministeriais	Articulação entre o Ministério da Cidadania e o Ministério da Saúde para o enfrentamento da tuberculose, especialmente às pessoas em situação de vulnerabilidade social ^( 33 , 41 , 45 , 47 )^ .	
		Articulação interministerial ^( 6 , 33 , 35 )^ e atuação do CIEDDS*** no âmbito do Ministério da Saúde ^( 40 , 42 , 44 )^ .	
		Articulações intersetoriais com áreas estratégicas como a Atenção Básica, Saúde Indígena, Saúde Mental, Saúde do Trabalhador e atuação multissetorial por meio da Rede Brasileira de Comitês para o Controle da Tuberculose, Rede Brasileira de Enfermagem pelo Fim da Tuberculose como Problema de Saúde Pública, da Parceria Brasileira de Luta contra a Tuberculose e da Frente Parlamentar de Luta Contra a Tuberculose ^( 6 )^ .	
	Celeridade da justiça	Prioridade na tramitação de processos judiciais e administrativos nos quais figure como parte ou interessada a pessoa acometida por tuberculose ^( 26 , 30 )^ .	

*SUS = Sistema Único de Saúde; ^†^SUAS = Sistema Único de Assistência Social; ^‡^Centro Pop = Centro de Referência Especializado para População em Situação de Rua; ^§^FGTS = Fundo de Garantia do Tempo de Serviço; ^||^CRAS = Centro de Referência da Assistência Social; ^¶^CREAS = Centro de Referência Especializado da Assistência Social; **BPC = Benefício de Prestação Continuada; ^††^PBF = Programa Bolsa Família; ^‡‡^INSS = Instituto Nacional do Seguro Social; ^§§^PIS/PASEP = Programa de Integração Social e ao Programa de Formação do Patrimônio do Servidor Público; ^||||^RAS = Rede de Atenção à Saúde; ^¶¶^BCG = Bacilo de Calmette e Guérin; ***CIEDDS = Comitê Interministerial para a Eliminação da Tuberculose e de Outras Doenças Determinadas Socialmente

## Discussão

No contexto brasileiro, considerando a importância das pessoas acometidas por TB serem consideradas como sujeitos em sua integralidade, as políticas de proteção social possuem a perspectiva de promover o acesso da população aos seus direitos fundamentais, de forma a garantir o exercício da cidadania, o atendimento às necessidades básicas e a promoção da dignidade humana^(31)^. Além disso, materializam a importância legal, ética e moral diante da eficácia de sua resposta nos desfechos do tratamento da TB e alívio do sofrimento entre as pessoas e famílias afetadas pela doença^(48)^.

Um estudo aponta que a disponibilidade e distribuição gratuita do medicamento antituberculose não são suficientes para a continuidade do tratamento da TB ou a cura, visto que a adesão ao tratamento não se reduz a uma vontade de cunho exclusivamente individual, mas se associa a outras dimensões transversais aos processos de produção e reprodução social^(49)^. Isto sugere uma abordagem coordenada que inclua não apenas serviços de saúde, mas também uma articulação intra e intersetorial baseada em um forte sistema de proteção social.

Neste sentido, faz-se necessário repensar políticas públicas que representem pautas próprias e compreender o que elas traduzem, sua construção histórica e as relações de poder estabelecidas, que levem em conta os anseios e as necessidades da população. Isso porque as instituições de poder não se preocupam de fato com as políticas enquanto um instrumento social, a não ser aquelas de cunho meramente assistenciais que apenas atenuam as desigualdades sociais^(50)^.

Após a promulgação da Constituição Federal de 1988, que apresenta o sistema de seguridade social brasileiro, a interseção entre saúde e direitos humanos foi revitalizada com a inclusão da saúde como “*direito de todos e dever do Estado*”^(15)^. A partir desse contexto, identificou-se a construção de um caminho heterogêneo das políticas públicas para a proteção social às pessoas acometidas por TB no país, marcado por diferentes períodos políticos, sociais e ideológicos, com a retomada de visibilidade somente após 21 anos da promulgação da Carta Magna brasileira e da alteração da legislação referente à declaração do imposto de renda^(27)^, com uma lei voltada à celeridade de processos judiciais e administrativos, que incluiu as pessoas com TB^(26)^.

Posteriormente a esse tímido cenário, o ano de 2019 foi um importante marco para a discussão da TB enquanto problema de saúde pública cujo enfrentamento ultrapassa o âmbito da saúde. Naquele ano foram publicados dois documentos normativos - o Manual de Recomendações para o Controle da TB no Brasil^(32)^ e a Instrução Operacional Conjunta nº 1/2019^(47)^, os quais sustentam e norteiam a maioria das políticas públicas voltadas à proteção social que foram identificadas neste estudo, uma vez que apresentam estratégias para o fortalecimento da articulação intra e intersetorial para garantia dos direitos humanos e cidadania nas ações de prevenção e cuidado da TB^(32,47)^.

A partir disso, para o alinhamento ao Pilar 2 da estratégia *The End TB*, voltado às políticas arrojadas e aos sistemas de apoio, a TB começa a ser pautada com mais força nas agendas políticas das esferas governamentais brasileiras, por meio de documentos normativos articulados com os Poderes Executivo e Legislativo, bem como nas agendas de trabalho da saúde, previdência e assistência social que possibilitam a proteção social às pessoas acometidas por TB^(32)^. Nessa perspectiva, as ações de prevenção e cuidado da TB devem ser orientadas pelo enfrentamento das desigualdades sociais e iniquidades em saúde que incidem sobre as pessoas acometidas pela doença e seus familiares, visando à ruptura de ciclos de pobreza e miserabilidade, bem como a sustentabilidade na geração de emprego e renda^(2)^.

Estima-se que o adoecimento e as mortes por TB podem causar um grave impacto na economia global de aproximadamente 1 trilhão de dólares entre 2015 e 2030^(51)^. Por outro lado, um estudo discute que estratégias de proteção social de base governamental, com um sólido compromisso político e recursos financeiros adequados, apresentam maior potencial para o alcance da meta global de 85% de sucesso no tratamento da TB^(52)^. Para isso, uma das questões primordiais refere-se à garantia de investimento público, de forma que tanto o orçamento centralizado do Ministério da Saúde quanto os recursos descentralizados por meio de fundos estaduais ou municipais sejam ampliados pelas três esferas da gestão brasileira^(42)^.

Este cenário só é possível a partir de políticas públicas que interfiram na produção da saúde no território, como aquelas voltadas à segurança alimentar e nutricional, habitação, saneamento, água, emprego, renda, educação, transporte, entre outras^(40)^, reencontrando o conceito ampliado de saúde preconizado na Constituição Federal e na Lei Orgânica da Saúde^(17)^.

Também foi possível identificar o protagonismo do SUS frente às ações de prevenção e cuidado integral às pessoas acometidas por TB, considerando a transversalidade do direito à saúde. No entanto, apesar de o SUS oferecer tratamento universal e gratuito^(32)^, compreende-se que a TB promove prejuízos e agravamento da situação socioeconômica, o que pode desestabilizar a dinâmica familiar por gerar gastos adicionais com alimentação, transporte, outros medicamentos e exames, por exemplo^(39,47)^.

Esses gastos, diretos ou indiretos, quando ultrapassam 20% da renda familiar anual, são considerados custos catastróficos gerados pelo adoecimento da TB^(53)^ e impactam diretamente na adesão e no desfecho do tratamento da doença, além de ocasionar sequelas sociais^(54)^. Para mitigar tais custos ou até mesmo, de forma audaciosa, alcançar a meta de que nenhuma pessoa com TB precise arcar com custos catastróficos ou repercussões sociais pela doença^(3)^, faz-se necessário garantir que as pessoas com TB e as famílias afetadas tenham acesso a intervenções de proteção social contra riscos financeiros decorrentes do tratamento^(55)^.

Uma das propostas para esta situação é o Projeto de Lei (PL) nº 6.991, de 2013, que visa garantir apoio financeiro às famílias em situação de pobreza que tenham, entre seus componentes, pessoas acometidas por TB, por meio do pagamento de um benefício financeiro no valor de meio salário mínimo durante o tempo em que durar o tratamento da TB^(56)^. Este PL, mesmo após 10 anos de sua elaboração, ainda se encontra em tramitação na Câmara dos Deputados, o que pode indicar uma falta de prioridade para a aprovação de legislações que tratam de transferência condicionada de renda para enfrentamento de doenças determinadas pela pobreza, tal como a TB.

Planejar e implementar políticas de saúde em coesão com políticas sociais pode ser altamente eficaz em países com alta carga para a TB. Um exemplo exitoso disso na Região das Américas é a Argentina, que, embora não esteja na lista dos 30 países nesta situação, implementou a política de transferência condicionada de renda para as pessoas acometidas por TB por meio do Decreto nº 170/91 da Lei 10.436 de financiamento estatal para a efetivação do pagamento de um salário mínimo a todas as pessoas elegíveis vinculadas ao Programa Provincial de Controle da TB. Esta lei de proteção econômica foi fundamental para motivar e acompanhar os casos de difícil manejo, aumentando a adesão ao tratamento da TB^(57-58)^.

É importante ressaltar que os direitos das pessoas acometidas pela TB são os mesmos que aqueles referentes ao conjunto da população, sem distinção, embora existam situações e condicionalidades para acesso a direitos específicos no âmbito da saúde, assistência e previdência social. Neste sentido, identificou-se neste estudo um conjunto de medidas que abrangeram políticas públicas voltadas à inclusão social, ao enfrentamento da pobreza e à garantia de acesso aos direitos sociais, à segurança alimentar e nutricional, transporte, trabalho, moradia e benefícios previdenciários às pessoas acometidas por TB.

Algumas dessas estratégias de proteção social são mais focalizadas durante o tratamento da TB e são caracterizadas como “ações específicas para a TB”, as quais beneficiam as pessoas acometidas pela doença e seus familiares e são incorporadas aos programas de tratamento da TB existentes. Outras fazem parte de um esquema ampliado de seguridade social, possuindo grande potencial na modificação de condições estruturais da sociedade, por fortalecer a resiliência econômica, o alívio da pobreza e agir em outros determinantes sociais intrínsecos ao adoecimento e à continuidade da cadeia de transmissão da TB^(59)^.

Estudo realizado no Brasil identificou as potencialidades na implementação de ações específicas para a TB, a exemplo do fornecimento de vale-alimentação às pessoas acometidas por TB, o que elevou em 13% a taxa de cura em comparação com o grupo sem tal intervenção^(60)^. Já em relação às ações sensíveis para a TB, estudos apresentaram um dos maiores exemplos brasileiros: o Programa Bolsa Família (PBF), que, por meio de transferência condicionada de renda, foi responsável por uma taxa de cura de 7,6%^(61)^ e 8%^(62)^ maior e uma perda de acompanhamento 7% menor em grupos beneficiários do PBF com TB^(61)^.

No entanto, é importante compreender os desafios que perpassam a consolidação dessas políticas no enfrentamento da TB. Para a garantia dos direitos analisados neste estudo, o acesso às políticas de proteção social perpassou condicionalidades, a exemplo da necessidade de documentação civil, vinculação ao Cadastro Único para acesso ao SUAS ou obrigatoriedade de contribuição ao INSS para acesso aos benefícios da previdência social, o que pode ocasionar barreiras para a efetivação dos direitos, sobretudo para populações em situação de maior vulnerabilidade social, como as pessoas em situação de rua.

Isso porque, além dessa população ser considerada a mais vulnerável ao adoecimento por TB, com um risco 56 vezes maior quando comparado ao da população geral do país^(30)^, evidencia-se a discriminação e invisibilidade das quais tais pessoas são alvo, seja pela falta de documentos, de domicílio, por estereótipo ou pelo uso de drogas, o que reforça a desassistência, as privações e o cerceamento de direitos, da cidadania e da própria condição humana^(63)^.

Durante a pandemia de Covid-19, houve indícios de que essas questões tenham se intensificado ainda mais, considerando a sobreposição de vulnerabilidades sociais e programáticas e o próprio impacto das medidas restritivas causadas na sociedade e nas ações de prevenção e cuidado da TB^(64-65)^. Para o enfrentamento de barreiras de acesso aos direitos e de ações de cuidado integral à saúde, alguns documentos normativos foram elaborados nesse período visando à proteção social dessas pessoas, com ênfase na articulação e no compartilhamento do cuidado entre as equipes do Centro Pop e dos Consultórios na Rua^(37,39)^.

As normativas voltadas para o enfrentamento do estigma e discriminação da TB no país acompanham um movimento de combate às concepções que marcaram a vivência da TB no passado e que até hoje impõem restrições e entraves ao tratamento, visto que complexificam a trajetória de cuidado e marginaliza as pessoas acometidas pela doença^(30,47)^. Assim, documentos que apresentam medidas direcionadas a esse tipo de combate possibilitam o resgate do direito a situações não humilhantes, degradantes ou de ofensa à dignidade humana^(48)^.

A atuação intersetorial, tal como entre o SUS e o SUAS, e a articulação interministerial por meio do Comitê Interministerial para a Eliminação da Tuberculose e de Outras Doenças Determinadas Socialmente (CIEDDS)^(44)^ avançam na perspectiva da priorização de estratégias que promovam proteção social. Essa priorização pode ser efetivada mediante um sinergismo entre áreas estratégicas ministeriais para o enfrentamento dos determinantes sociais da TB nas áreas de assistência social, justiça e segurança pública, trabalho e renda, direitos humanos, igualdade racial, povos originários, educação e cidadania^(40,42,44)^. Além disso, cabe ressaltar a importância da responsabilidade social e do envolvimento de áreas como a enfermagem, que pode contribuir substancialmente para a redução das desigualdades sociais e de saúde subjacentes de diferentes contextos comunitários^(66)^.

Enquanto limitações deste estudo, é importante destacar a atualização das plataformas de busca, que, embora tenham recuperado um volume robusto de informações, apresentam fragilidades quanto à disponibilização de materiais, e alguns documentos normativos podem não ter sido identificados por não estarem disponíveis eletronicamente. Além disso, as discussões suscitadas possuem um teor crítico-reflexivo dos pesquisadores e podem não ter abrangido outras interpretações também cabíveis na arena de disputa das políticas públicas brasileiras, com o recorte analítico da proteção social às pessoas acometidas pela TB.

Por fim, os resultados desta pesquisa documental avançam no conhecimento científico no campo da saúde pública ao ampliar a compreensão sobre as complexas interações entre o direito à saúde, os direitos sociais e a justiça social no contexto do enfrentamento da TB, fornecendo contribuições para orientar a formulação e implementação de políticas públicas mais eficazes e inclusivas destinada a reduzir as desigualdades sociais e de saúde que atravessam fortemente as pessoas acometidas por TB e seus agregados familiares.

## Conclusão

Este estudo identificou e analisou documentos normativos que procuram garantir o direito à proteção social por meio de políticas públicas no enfrentamento da TB que abrangeram o direito à saúde, à assistência e à previdência social, bem como ações que envolvem o compartilhamento de responsabilidades para efetivação de tais direitos.

Tais políticas são consideradas recentes no histórico do Estado Democrático de Direito e possuem ainda um longo caminho para uma abordagem abrangente, centrada nas pessoas acometidas pela TB e que sejam, de fato, baseadas em direitos para a prevenção, o cuidado e o apoio por meio de articulações intersetoriais e interministeriais, com forte atuação do SUS e do SUAS.

Entretanto, os desafios impostos pela dificuldade ou ausência de acesso a esses direitos, associada a outras situações que potencializam a vulnerabilidade social, fazem com que as pessoas acometidas por TB continuem desprotegidas socialmente. Neste sentido, cabe reafirmar o papel e dever do Estado em garantir a proteção social por meio de políticas públicas que promovam a vida, a cidadania, os direitos humanos e a justiça social.

# Translation: Políticas públicas de protección social para personas afectadas por tuberculosis: un análisis documental*

**DOI:** 10.1590/1518-8345.7526.4502

**Authors:** Melisane Regina Lima Ferreira, Jaqueline Garcia de Almeida Ballestero, Rubia Laine de Paula Andrade, Tiemi Arakawa, Inês Fronteira, Aline Aparecida Monroe

**Affiliations:** 1 Universidade de São Paulo, Escola de Enfermagem de Ribeirão Preto, Centro Colaborador de la OPS/OMS para el Desarrollo de la Investigación en Enfermería, Ribeirão Preto, SP, Brasil; 2 Becaria de la Coordenação de Aperfeiçoamento de Pessoal de Nível Superior (CAPES), Brasil; 3 Ministério da Saúde, Coordenação Geral de Vigilância da Tuberculose, Micoses endêmicas e Micobactérias não tuberculosas, Departamento de HIV/Aids, Tuberculose, Hepatites Virais e Infecções Sexualmente Transmissíveis, Brasília, DF, Brasil; 4 Universidade NOVA de Lisboa, Escola Nacional de Saúde Pública, Lisboa, Portugal; 5 Becaria del Conselho Nacional de Desenvolvimento Científico e Tecnológico (CNPq), Brasil

**Keywords:** Tuberculosis, Política Pública, Bienestar Social, Política de Salud, Derechos Humanos, Seguridad Social

## Abstract

analizar los documentos normativos que buscan garantizar el derecho a la protección social de las personas afectadas por tuberculosis vigentes en Brasil en el 2023.

investigación documental cualitativa, realizada en septiembre del 2023, con base en el levantamiento de documentos en el ámbito nacional, estatal y municipal, provenientes de órganos gubernamentales e instancias de control social tras la promulgación de la Constitución Federal, en cuatro plataformas electrónicas, exportados y organizados en el programa informático Atlas.ti, e interpretados con base en análisis de contenido, modalidad temática.

el *corpus* analítico estuvo compuesto por 30 documentos normativos, a saber: nueve leyes, siete materiales técnico-institucionales, cinco ordenanzas, cuatro resoluciones, dos decretos, un convenio de cooperación técnica, una instrucción normativa y una instrucción operativa, de los cuales surgieron cuatro categorías temáticas: el derecho a la salud, el derecho a la asistencia social, el derecho a la seguridad social y el reparto de responsabilidades.

las políticas de protección de personas con tuberculosis en Brasil son recientes y aún queda un largo camino que recorrer para un abordaje integral por medio de articulaciones intersectoriales e interministeriales, con el objetivo de afrontar la vulnerabilidad social y reafirmar el deber del Estado de garantizar la protección social mediante políticas públicas que promuevan la vida, la ciudadanía, los derechos humanos y la justicia social.

## Destacados

**(1)** El derecho a la salud incluye políticas públicas en el SUS para el afrontamiento de la TB. **(2)** El derecho a la asistencia social ha destacado el acceso a servicios, programas y beneficios. **(3)** El derecho a la seguridad social ha cubierto pensiones y ayudas para las personas con TB. **(4)** Responsabilidad interministerial, intersectorial y compartida entre el SUS y el SUAS. **(5)** Persisten los desafíos en el acceso a los derechos sociales y de salud por parte de las personas con TB.

## Introducción

La tuberculosis (TB) es una de las enfermedades más emblemáticas relacionadas con la pobreza y fuertemente influenciada por determinantes sociales. A la vez, se considera perpetuadora de ciclos de miseria debido a los impactos sociales y económicos que genera la enfermedad^(1)^. Como enfermedad multicausal, la TB requiere una respuesta multisectorial y sinergia entre políticas públicas de protección social que promuevan la eliminación de la pobreza, la equidad, la justicia y los derechos humanos de las personas afectadas por la enfermedad, incluido el combate a todas las formas de discriminación y estigma^(2)^.

La estrategia *The End TB,* de la Organización Mundial de la Salud (OMS), recomienda una reducción del 90% en las tasas de incidencia y del 95% en las tasas de mortalidad para el 2035 para eliminarla como endémica para el 2050, con el objetivo adicional de que ninguna persona con TB deba soportar costos catastróficos o repercusiones sociales debido a la enfermedad^(3)^. En consonancia con los Objetivos de Desarrollo Sostenible (ODS) de las Naciones Unidas, la TB está incluida en el Objetivo 3.3 del tercer ODS, cuya propuesta es reducir el 90% de las muertes por TB y el 80% de los nuevos casos de la enfermedad para el 2030. Este objetivo también está estrechamente relacionado con el décimo objetivo relativo a la reducción de las desigualdades^(4)^.

Sin embargo, en el 2022, se estimó que la tuberculosis habría afectado a 10,6 millones de personas en todo el mundo, con 1,3 millones de muertes a causa de la enfermedad y otras 167.000 muertes entre personas que viven con VIH/sida. En el mismo año, la región de las Américas fue responsable del 3,1% del total de casos de TB en todo el mundo y de un número estimado de muertes causadas por la enfermedad mucho mayor que en el 2015 (+41%)^(3)^. En esta región, solo Brasil está incluido en la lista de los 30 países con mayores cargas de tuberculosis en el ámbito mundial, ya que, en el 2022, el país fue responsable de 78.057 nuevos casos de TB, con un coeficiente de incidencia de 36,3 casos/100.000 habitantes, además de 5.162 muertes por la enfermedad, con un coeficiente de mortalidad de 2,2 muertes/100.000 habitantes^(5)^.

Esto implica decir que el país aún está lejos de lograr las propuestas de la estrategia *The End TB* y del Plan Nacional por el Fin de la TB como problema de salud pública, que recomiendan para el 2023 reducir la tasa de incidencia de la enfermedad a menos de 10 casos/100.000 habitantes y una muerte/100.000 habitantes^(6-7)^. Considerando este escenario, una de las propuestas más audaces para el fin de la TB se basa en el 2.º Pilar relativo a las políticas audaces y a los sistemas de apoyo, aspirando a la protección social de las personas afectadas por la enfermedad, además de reducir la pobreza y otros factores determinantes para la enfermedad por TB^(3)^.

La protección social se refiere a acciones que implican políticas públicas que buscan garantizar la vida y los derechos humanos, prevenir la incidencia de riesgos y vulnerabilidades, reducir daños e impactos negativos resultantes de restricciones sociales, económicas, políticas, naturales o de ofensas a la dignidad humana^(8)^. Para afrontar la TB, una revisión de literatura realizada desde una perspectiva mundial mostró que las medidas y estrategias dirigidas a la protección social como un derecho de las personas afectadas por la TB mejoran el estado nutricional, la calidad de vida y la adhesión al tratamiento, reduciendo costos catastróficos y promoviendo resultados favorables del tratamiento^(9)^.

Además, un estudio que analizó mundialmente la asociación entre el gasto en protección social y la carga de TB mostró que los países que invierten una parte elevada de su Producto Interno Bruto (PIB) en políticas de protección social experimentan tasas más bajas de prevalencia, incidencia y mortalidad por TB^(10)^. Tales cuestiones ponen de manifiesto la alineación de la protección social con las políticas públicas propuestas para superar el desafío de eliminar la TB como un problema de salud pública a nivel nacional e internacional.

Sin embargo, aún existen brechas en la comprensión de cómo se regulan las medidas y estrategias de protección social como políticas públicas, así como la manera en que se orientan para su implementación en diferentes contextos socioeconómicos. Con base en esto, este estudio pretende contribuir al avance del conocimiento científico explorando cómo se normalizan las políticas de protección para las personas con TB en el ámbito nacional y cómo se pueden adaptar de una manera más inclusiva para mejorar los mecanismos de apoyo y tratamiento disponibles, impactando la calidad de vida de las personas afectadas por la enfermedad y la eficiencia de los sistemas de salud.

En ese sentido, las políticas públicas encaminadas a superar las desigualdades que repercuten en la salud humana y que en sí mismas se caracterizan como injustas son fundamentales para lograr una sociedad más equitativa y, por lo tanto, tienen un impacto en el control de la TB^(11)^. Por lo que es oportuno analizar la coyuntura teniendo en cuenta las potencialidades de Brasil y los desafíos futuros que la crisis financiera y las políticas de austeridad pueden fomentar en el sistema brasileño de salud y asistencia social.

Así, guiados por la pregunta de investigación “¿cómo se incluye la protección social de las personas afectadas por la TB en el marco de las políticas públicas brasileñas?”, el objetivo fue analizar los documentos normativos que buscan garantizar el derecho a la protección social de las personas afectadas por TB vigentes en Brasil en el 2023.

## Método

### Diseño del estudio

Se trata de una investigación documental cualitativa, realizada a partir de documentos que regulan las políticas públicas que incluyen en su alcance medidas de protección social para personas afectadas por TB en el escenario nacional brasileño. Este tipo de investigación se caracteriza por la búsqueda de información en documentos con datos originales basándose en métodos y técnicas para la captación, comprensión y análisis de información factual por parte del investigador, ya que los documentos no han sido sometidos previamente a ningún tratamiento científico^(12-13)^.

El uso de documentos en la investigación permite recuperar un caudal de información que posibilita ampliar el entendimiento de temáticas cuya comprensión requiere una contextualización histórica y sociocultural^(12)^. En ese sentido, los documentos oficiales se consideran fuentes fidedignas de datos, ya que permiten un análisis contextual de actos normativos y políticos^(14)^.

### Criterios de selección

Se decidió incluir documentos posteriores al hito histórico-temporal de la Constitución Federal de 1988, que garantiza la protección social en Brasil mediante el trípode formado por las políticas de salud, de asistencia social y de seguridad social, que constituyen la seguridad social en el país^(15)^. Con base en este contexto, se realizó el levantamiento de material técnico institucional, así como de legislaciones, decretos, ordenanzas, resoluciones y demás actos normativos nacionales, estatales o municipales, provenientes de órganos gubernamentales y de sus instancias de control social, que se encuentran vigentes.

Se excluyeron documentos relacionados con la incorporación de nuevos regímenes terapéuticos antituberculosis, pruebas diagnósticas de TB, consultas públicas, proyectos de ley, documentos establecidos por organismos internacionales, así como normativas que no especificaran medidas de protección social dirigidas a las personas afectadas por TB.

### Levantamiento documental

Las búsquedas fueron realizadas por la investigadora principal, en septiembre del 2023, en las siguientes plataformas electrónicas: Diario Oficial de la Unión (DOU) (https://www.in.gov.br/consulta/) para documentos federales, estatales o municipales; Portal de la Legislación Federal Brasileña (https://legislacao.presidencia.gov.br/) para documentos federales; Portal de las Leyes Estatales (https://leisestaduais.com.br/) para documentos estatales o municipales; y el sitio *web* oficial de publicaciones del Ministerio de la Salud (https://www.gov.br/saude/pt-br/centrais-de-conteudo/publicacoes) para documentos federales, utilizando las palabras clave: “Tuberculosis” y “Protección Social”.

Para las búsquedas en el DOU, con el objetivo de optimizar el proceso de levantamiento documental, se utilizó el filtro “Organización Principal” para seleccionar documentos emitidos por órganos con competencia directa sobre las políticas de protección social y “Tipo de Acto” para seleccionar actos específicos que establecen o regulan políticas y prácticas relacionadas con la protección social, con relevancia normativa y legal. Además, para las consultas de documentos con fecha anterior al 01/01/2018 se utilizaron los filtros “Buscar en la Versión Certificada” o “Diario Completo Certificado”. En el caso del sitio *web* del Ministerio de Salud, se utilizó el filtro “todas las publicaciones”, y para las demás plataformas, se utilizaron los filtros de “normas en vigor” o “publicadas” y un recorte temporal a partir de 1988.

Cabe señalar que, con el fin de reducir un posible sesgo de intereses en el uso del Portal de Leyes Estatales, se adoptaron medidas en materia de transparencia en la elección de la legislación estatal y municipal, así como un análisis riguroso de los datos, para evitar distorsiones y preservar la integridad y la imparcialidad del presente estudio. También se resalta que no se incluyeron documentos de sitios web de la sociedad civil organizada, ya que el objetivo del estudio estaba relacionado con examinar las políticas implementadas y no las demandas de los diferentes actores sociales.

### Tratamiento y análisis de datos

Tras el levantamiento y acceso a las publicaciones oficiales, los documentos se guardaron en formato PDF y se exportaron al programa informático Atlas.ti, versión 23, con el objetivo de organizarlos e interpretarlos, mediante análisis de contenido, modalidad temática que se desarrolló en tres etapas, según los supuestos de Bardin^(16)^, a saber: preanálisis documental; exploración del material; y tratamiento de los resultados. En la etapa de preanálisis, realizada íntegramente por dos investigadoras, incluida la principal, se realizó la lectura de los textos, para identificar su relación con el objetivo del estudio, y la formulación de núcleos de significado con base en cinco dimensiones^(14)^: análisis del contexto; de la autoría; intereses o confiabilidad del texto; naturaleza del texto; y conceptos clave del texto.

Luego, se procedió a la exploración del material mediante la lectura en profundidad de los documentos que constituyeron el *corpus* analítico. Para la extracción de la información de dichos documentos realizada por la investigadora principal, se utilizó una hoja de cálculo de *MicrosoftExcel* para codificarlos y categorizarlos según la tipología, el año de publicación, el organismo o la institución de origen, el alcance y los tópicos o temas abordados. Estas categorías fueron certificadas por otras dos investigadoras, cuya etapa de certificación incluyó la revisión y validación de las categorías con relación a los datos extraídos y al cumplimiento del objeto del estudio.

En la etapa de procesamiento de los resultados, se realizó estadística descriptiva simple de los datos relativos a la categorización de los documentos. Finalmente, los hallazgos fueron interpretados por las tres investigadoras directamente involucradas en las etapas anteriores (incluida la principal), de acuerdo con los cuestionamientos del estudio para analizarlos desde la perspectiva del derecho a la protección social, que abarca medidas encaminadas a garantizar la salud, la asistencia y la seguridad social. Cabe señalar que este estudio se basa en una concepción ampliada de la salud propuesta por el movimiento de Reforma Sanitaria brasileña y en la noción de derecho como logro social, y es deber del Estado proporcionar las condiciones indispensables para el pleno ejercicio de la salud y asegurar el acceso universal e igualitario a las acciones y a los servicios para su promoción, protección y recuperación^(17)^.

### Aspectos éticos

Si bien no es necesaria la aprobación ética para realizar una investigación documental, cabe señalar que este estudio forma parte de una investigación sobre la evaluación de la protección social de las personas afectadas por TB, aprobada por el Comité de Ética en Investigación de la institución responsable, bajo el dictamen número 6.389.278, de 05/10/2023 (CAAE 71246023.6.0000.5393). Además, buscamos utilizar un lenguaje no estigmatizante relacionado con la TB durante todo el estudio^(18)^.

## Resultados

El levantamiento documental en las plataformas electrónicas seleccionadas en este estudio resultó en la inclusión de 30 documentos normativos publicados por medio de nueve leyes (30%)^(19-27)^, siete materiales técnico-institucionales (23,4%)^(6,28-33)^, cinco ordenanzas (16,7%)^(34-38)^, cuatro resoluciones (13,3%)^(39-42)^, dos decretos (6,7%)^(43-44)^, un convenio de cooperación técnica (3,3%)^(45)^, una instrucción normativa (3,3%)^(46)^ y una instrucción operativa (3,3%)^(47)^, en el período comprendido entre 1988 y agosto del 2023, y el 2022 fue el año con el mayor número de publicaciones (n= 08, 26,6%) (Figura 1).

**Figura 1 -:** Línea histórica representativa de los rumbos de las políticas públicas de protección social de las personas afectadas por TB en Brasil

*PRES/INSS = Peritaje Médico del Instituto Nacional de Seguridad Social; ^†^DIRBEN/INSS = Dirección de Beneficios del Instituto Nacional de Seguridad Social; ^‡^MTP = Ministerio de Trabajo y Seguridad Social; ^§^MS = Ministerio de Salud

Las normas incluidas que regulan u orientan la protección social de las personas afectadas por TB (Figura 2) provinieron en su mayoría de órganos gubernamentales de alcance nacional (n=22, 73,3%), tales como la Casa Civil^(21-22,26-27,43-44)^, el Ministerio de Derechos Humanos y Ciudadanía^(36-39,45,47)^, el Ministerio de Salud^(28-33)^ y el Ministerio de Trabajo y Empleo^(34-35,46)^; estatales (n= 05, 16,7%), como la Asamblea Legislativa de Mato Grosso^(20)^ y gobiernos estatales de Río de Janeiro^(23,25,41)^ y Rio Grande do Norte^(19)^; y cobertura municipal (n=01, 3,3%), por medio de la Cámara Municipal de Río de Janeiro^(24)^. Además, dos publicaciones (6,7%) provinieron del Consejo Nacional de Salud^(40,42)^.

**Figura 2 -:** Caracterización de los documentos normativos que buscan garantizar el derecho a la protección social de las personas afectadas por TB, en cuanto a su nombre, definiciones y articulación con la protección social de las personas afectadas por TB (n = 30)

**Nombre del documento**	**Definiciones y articulación con la protección social de las personas afectadas por TB**
Convenio de Cooperación Técnica n.º 60/2021 ^( 45 )^	- Integra las políticas públicas de salud con la asistencia social;
	- Establece beneficios sociales para personas en situación de vulnerabilidad social y más susceptibles a la tuberculosis;
	- Desarrolla programas específicos que abordan las necesidades de las personas con tuberculosis.
Decreto n.º 10.410, de 30 de junio del 2020 ^( 43 )^	- Ajusta las reglas de acceso a los beneficios de la seguridad social, como subsidio por enfermedad, jubilación por invalidez y otros beneficios que son esenciales para los trabajadores incapacitados temporal o permanentemente debido a la tuberculosis.
	- Contribuye a la prevención de la vulnerabilidad socioeconómica de las personas afectadas por tuberculosis.
Decreto n.º 11.494, de 17 de abril del 2023 ^( 44 )^	- Establece un comité que coordina los esfuerzos interministeriales para enfrentar los determinantes sociales de la tuberculosis;
	- Integra e implementa políticas que protegen los derechos y el bienestar de las personas con tuberculosis.
Instrucción Normativa PRES/ INSS* n.º 128, de 28 de marzo del 2022 ^( 46 )^	- Establece procedimientos claros y accesibles para el acceso a los beneficios de la seguridad social, asegurando que las personas con tuberculosis reciban el apoyo financiero necesario durante el tratamiento y la recuperación.
	- Ofrece una red de seguridad financiera para las personas con tuberculosis y ayuda a mitigar el impacto económico de la enfermedad.
Instrucción Operativa Conjunta n.º 1, de 26 de septiembre del 2019 ^( 47 )^	- Promueve la colaboración entre la salud y la asistencia social necesaria para superar las barreras impuestas por la tuberculosis, como la pérdida de ingresos, la necesidad de apoyo alimentario, otras vulnerabilidades y la exclusión social.
Ley Complementaria n.º 570, de 26 de abril del 2016 ^( 19 )^	- Fortalece la protección social de las personas con tuberculosis al garantizarles acceso a una jubilación por invalidez con beneficios completos, brindándoles seguridad financiera y reconociendo la gravedad de la enfermedad en términos legales y de seguridad social.
Ley n.º 11.576, de 17 de noviembre del 2021 ^( 20 )^	- Crea una política estatal específica para el control y la eliminación de la tuberculosis mediante la coordinación con otras áreas, como asistencia social, vivienda y alimentación, que son fundamentales para la protección social de las personas afectadas.
Ley n.º 13.677, de 13 de junio del 2018 ^( 21 )^	- Proporciona acceso a recursos financieros que pueden utilizarse para satisfacer las necesidades emergentes durante el tratamiento de la tuberculosis y el alivio financiero, complementando otras formas de apoyo social y económico.
Ley n.º 14.289, de 3 de enero del 2022 ^( 22 )^	- Promueve la protección social al prevenir el estigma y la discriminación relacionados con la tuberculosis garantizando los derechos humanos y la dignidad y creando un entorno más seguro y confidencial para que las personas puedan buscar y continuar el tratamiento sin temor a una exposición indebida.
Ley n.º 6.541, de 19 de septiembre del 2013 ^( 23 )^	- Contribuye a la protección social al facilitar el acceso al tratamiento, reducir los costos asociados a la enfermedad, fomentar la continuidad del tratamiento y promover la inclusión social de las personas con tuberculosis.
Ley n.º 7.286, de 3 de marzo del 2022 ^( 24 )^	- Crea una política municipal específica para el control y la eliminación de la tuberculosis mediante la coordinación con otras áreas, como la asistencia social, la vivienda y la seguridad alimentaria, que son fundamentales para la protección social de las personas afectadas.
Ley n.º 8.746, de 9 de marzo del 2020 ^( 25 )^	- Crea una política estatal específica para el control de la tuberculosis mediante la garantía de los servicios de salud, la coordinación de las políticas públicas y la protección contra la discriminación y la exclusión social.
Ley n.º 12.008, de 29 de julio del 2009 ^( 26 )^	- Contribuye a la protección social de las personas con tuberculosis al facilitar el acceso rápido y eficiente a derechos y beneficios, reducir la burocracia y retrasos y brindar protección adicional y apoyo legal adecuado a quienes enfrentan la enfermedad.
Ley n.º 7.713, de 22 de diciembre de 1988 ^( 27 )^	- Al permitir exenciones y deducciones fiscales para las personas afectadas por la tuberculosis, puede reducir la carga financiera, aliviar los costos asociados al tratamiento y promover un apoyo económico crucial para el control de la enfermedad.
Asistencia del enfermero a la persona con tuberculosis en la Atención Primaria ^( 28 )^	- Vinculado a la protección social al asegurar el acceso a cuidados adecuados, promover la educación, realizar un seguimiento continuo, identificar y derivar a las personas con tuberculosis a tratamiento especializado, ofrecer apoyo psicosocial y coordinar con otros servicios.
Directrices Nacionales de articulación entre el Sistema Único de Salud y el Sistema Único de Asistencia Social para promover el cuidado integral y la protección social de las personas en situación de vulnerabilidad y riesgo social, que viven con VIH/sida, sífilis, hepatitis virales, lepra y tuberculosis ^( 29 )^	- Establece un marco para la integración de cuidados de salud y asistencia social;
	- Promueve un cuidado integral y asegura que las personas con tuberculosis reciban el apoyo que necesitan para afrontar sus complejas necesidades y mejorar su calidad de vida.
	- Afronta las vulnerabilidades adicionales, como dificultades financieras, estigma y falta de acceso a los servicios.
Guía orientadora: promoción de la protección social de las personas afectadas por la tuberculosis ^( 30 )^	- Proporciona directrices para que las personas con tuberculosis reciban un apoyo integral y eficaz, abordando sus necesidades de salud y sociales y promoviendo un sistema integrado de protección social;
	- Ofrece información sobre programas y políticas que tienen como objetivo reducir la carga financiera sobre las personas con tuberculosis y sus familias.
Línea de cuidado de la tuberculosis: orientaciones para gestores y profesionales de la salud ^( 31 )^	- Proporciona directrices para una atención coordinada, accesible y de alta calidad, además de ofrecer apoyo adicional que mejora la adhesión al tratamiento y a la calidad de vida de las personas con tuberculosis.
Manual de recomendaciones para el control de la tuberculosis en Brasil ^( 32 )^	- Establece directrices relacionadas con la protección social por medio del cuidado eficaz e integrado entre salud y asistencia social;
	- Orienta la educación y la capacitación de los profesionales de la salud y asegura que las personas con tuberculosis tengan acceso a los recursos y apoyo necesarios para afrontar la enfermedad y sus consecuencias.
Plan nacional por el fin de la tuberculosis como problema de salud pública: estrategias para 2021-2025 ^( 6 )^	- Establece un enfoque estratégico e integrado entre las políticas de salud y las políticas de protección social para el control de la enfermedad;
	- Recomienda acciones para el acceso equitativo al tratamiento, reduciendo los costos financieros y mejorando el apoyo social y los recursos disponibles para las personas con tuberculosis.
Red brasileña de comités para el control de la tuberculosis en Brasil: pasado, presente y futuro ^( 33 )^	- Aborda la coordinación de esfuerzos entre diferentes entidades y niveles de gobierno para el desarrollo de políticas y prácticas de protección social;
	- Comités bien estructurados y operativos ayudan a asegurar que las personas con tuberculosis tengan acceso a los cuidados, el apoyo social y los recursos que necesitan para afrontar la enfermedad.
Ordenanza DIRBEN/INSS ^†^ n.º 992, de 28 de marzo del 2022 ^( 34 )^	- Normaliza el acceso a los beneficios de la seguridad social, como la jubilación por invalidez o la ayuda por enfermedad;
	- Garantiza que las personas con tuberculosis reciban el apoyo financiero que necesitan para compensar la pérdida de ingresos debido a la incapacidad para trabajar.
Ordenanza interministerial MTP ^‡^ /MS ^§^ n.º 22, de 31 de agosto del 2022 ^( 35 )^	- Define directrices para la colaboración entre los ministerios responsables de la salud y el trabajo, asegurando un enfoque coordinado que aborde las necesidades de salud, las cuestiones de empleo y la protección social de las personas con tuberculosis de forma integrada y eficaz.
Ordenanza n.º 100, de 14 de julio del 2020 ^( 36 )^	- Garantiza que se continúen brindando servicios socioasistenciales esenciales durante las emergencias de salud pública, asegurando la continuidad del cuidado y la adaptación de los servicios a las nuevas condiciones, promoviendo apoyo tanto al manejo de la tuberculosis como para la protección social integral.
Ordenanza n.º 54, de 1 de abril del 2020 ^( 37 )^	- Garantiza la continuidad de los servicios de asistencia social durante contextos de crisis de salud pública, centrándose en medidas de seguridad y salud para usuarios y profesionales, asegurando que las personas con tuberculosis reciban servicios críticos para la protección social, como asistencia financiera y apoyo social, sin comprometer su salud.
Ordenanza n.º 69, de 14 de mayo del 2020 ^( 38 )^	- Aborda las circunstancias de la población en situación de calle durante emergencias de salud pública, garantizando que estas personas, que a menudo enfrentan condiciones adicionales de vulnerabilidad, estén socialmente protegidas y reciban la atención necesaria para el manejo de sus condiciones de salud, incluida la tuberculosis.
Resolución n.º 40, de 13 de octubre del 2020 ^( 39 )^	- Asegura que los derechos humanos y las necesidades específicas de la población sin hogar afectada por la tuberculosis se cumplan de manera integrada e integral por medio de políticas públicas alineadas para protegerlos.
Resolución n.º 719, de 17 de agosto del 2023 ^( 40 )^	- Define directrices y propuestas que pueden impactar directamente en las políticas de salud y en la asistencia social para mejorar la prevención, el tratamiento y el apoyo a las personas con tuberculosis.
Resolución n.º 2.580, de 23 de diciembre del 2021 ^( 41 )^	- Asegura la asignación de recursos para acciones específicas que tengan como objetivo mejorar el cuidado y el apoyo a las personas con tuberculosis;
	- Fomenta la colaboración entre la salud y la asistencia social y fortalece la capacidad de los municipios, promoviendo una protección social más sólida para la población afectada.
Resolución n.º 709, de 16 de marzo del 2023 ^( 42 )^	- Contribuye a un sistema de protección social más eficaz y holístico, garantizando que las personas con tuberculosis reciban el apoyo que necesitan para superar la enfermedad y mejorar su calidad de vida, mediante la integración de políticas de salud y asistencia social.

*PRES/INSS = Peritaje Médico del Instituto Nacional de Seguridad Social; ^†^DIRBEN/INSS = Dirección de Beneficios del Instituto Nacional de Seguridad Social; ^‡^MTP = Ministerio de Trabajo y Seguridad Social; ^§^MS = Ministerio de Salud

El análisis documental arrojó cuatro categorías temáticas que abarcaron sus respectivas unidades de sentido y los principales resultados identificados en los documentos normativos incluidos que conformaron el *corpus* del estudio (Figura 3). La primera categoría temática, la *derecho a la salud*, se refiere a documentos que presentaron políticas sociales y económicas para garantizar acciones de prevención y cuidado de la TB en el Sistema Único de Salud (SUS), de seguridad alimentaria y nutricional, transporte, trabajo, control social y afrontamiento del estigma y de la discriminación de la TB. Se resalta que en esta categoría también se identificaron documentos centrados específicamente en la protección social de las personas en situación de calle afectadas por TB.

La segunda categoría temática, la *derecho a la asistencia social*, incluyó documentos que presentaron el acceso a los servicios, programas, proyectos y beneficios socioasistenciales, derecho al Beneficio de Prestación Continua (BPC), a los beneficios eventuales, a la documentación civil, a los programas de transferencia de ingresos y a los servicios de recepción del Sistema Único de Asistencia Social (SUAS). El *derecho a la seguridad social* constituyó la tercera categoría temática que abarcó documentos que presentaron el derecho de las personas afectadas por TB a la jubilación por incapacidad permanente, a la ayuda por incapacidad temporal (ayuda por enfermedad), a la exención del impuesto a la renta y transacciones financieras vinculadas al Programa de Integración Social y al Programa de Formación del Patrimonial del Servidor Público (PIS/PASEP).

La última categoría temática incluyó documentos destinados a *compartir responsabilidades* en el afrontamiento a la TB, mediante las acciones del SUAS en conjunto con el SUS, acciones intersectoriales e interministeriales, así como normas encaminadas a acelerar la justicia para las personas afectadas por la TB.

**Figura 3 -:** Descripción del análisis documental respecto a las cuatro categorías temáticas, unidades de sentido y principales resultados encontrados en los documentos normativos que buscan garantizar el derecho a la protección social de las personas afectadas por tuberculosis (n = 30)

**Categorías temáticas**	**Unidades de sentido**	**Principales resultados (documentos normativos)**
*Derecho a la salud*	Acciones de prevención y cuidado de la tuberculosis en el SUS*	Atención integral a las necesidades de salud, económicas, psicológicas y sociales de las personas que padecen tuberculosis ^( 20 , 24 - 25 )^ , mediante la derivación a otras políticas públicas ^( 47 )^ .
		Consolidación y fortalecimiento de la línea de cuidado de la tuberculosis, con el fin de garantizar el acceso al cuidado y su continuidad entre los diferentes servicios y niveles de atención ^( 42 )^ .
		Identificación oportuna de personas con signos y síntomas de tuberculosis por parte de los equipos de salud y asistencia social ^( 30 )^ .
		Fortalecimiento y ampliación de las acciones de promoción de la salud por parte de los equipos y programas de Atención Primaria, con énfasis en la Estrategia de Salud de la Familia ^( 39 )^ .
		Articulación del Proyecto Terapéutico Singular del SUS* y del Plan Individual de Atención del SUAS ^†^ para la atención integral de la persona con tuberculosis y sus familias ^( 47 )^ .
		*Población en situación de calle afectada por tuberculosis*: articulación y reparto de cuidados entre los equipos del Centro Pop ^‡^ con los equipos de salud de los Consultorios en la Calle ^( 37^^.^^39 )^ .
	Seguridad alimentaria y nutricional	Garantía de una alimentación sana y adecuada ^( 24 - 25 , 29 , 40 )^ por medio de incentivos como meriendas ^( 28 )^ , leche ^( 28 )^ , distribución de canastas básicas de alimentos ^( 25 , 28 - 29 , 33 , 41 )^ , bono de alimentación o tarjeta alimentaria ^( 41 )^ .
		Acceso regular y permanente a alimentos de buena calidad y en cantidad suficiente, mediante el uso de restaurantes populares ^( 25 , 29 - 30 , 32 , 41 )^ , bancos de alimentos, comedores comunitarios, ferias y mercados populares ^( 30 )^ y de integración con el Sistema Nacional de Seguridad Alimentaria y Nutricional ^( 47 )^ .
	Transporte	Exención del pago de tasas por servicios de transporte intermunicipal ^( 23 , 25 , 30 , 32 )^ , mediante el otorgamiento de un máximo de 60 bonos sociales mensuales en un plazo máximo de 30 días laborales ^( 23 )^ o de 15 días laborales ^( 25 )^ .
		Garantía de bono social para el acompañante de la persona con tuberculosis que no pueda viajar sin acompañante ^( 25 , 32 )^ o que no sea capaz de moverse sola ^( 32 )^ .
		Acceso al transporte gratuito ^( 29 )^ mediante la facilitación de bonos de transporte ^( 28 )^ en ferries, metros, autobuses y trenes ^( 32 )^ .
	Trabajo	Programas para promover el acceso al mundo laboral ^( 29 )^ .
		Mantenimiento de las relaciones laborales mediante la prohibición del despido del trabajo por haber contraído tuberculosis, garantía de ambientes saludables y retiro del FGTS ^§(30)^ .
	Control social	Fomento a la participación de la representación de la sociedad civil, de representantes y liderazgos comunitarios y de personas afectadas por la tuberculosis en las instancias de control social del SUS* y SUAS ^†(29-30,33,45)^ .
		Establecimiento de foros de coordinación entre asistencia social y salud ^( 45 )^ .
	Combate al estigma y a la discriminación de la tuberculosis	Promoción de acciones de inclusión social de las personas con tuberculosis ^( 30 , 45 )^ , en las unidades de salud y socioasistenciales, en el territorio y en los espacios comunitarios ^( 6^^.^^47 )^ .
		Prohibición del sometimiento a tratos inhumanos o degradantes, privación de la libertad o de la convivencia familiar y discriminación por su morbilidad ^( 20 , 25 )^ .
		Obligación de preservar la confidencialidad sobre la condición de una persona con tuberculosis ^( 22 )^ .
		Orientación sobre situaciones discriminatorias, sobre el uso de canales de comunicación y registro de denuncias por medio de las defensorías o servicios como “Llama al 100”, “Llama al 180” y “Llama Salud 136” ^( 29 )^ .
*Derecho a la asistencia social*	Acceso a servicios, programas, proyectos y beneficios socioasistenciales	Garantía de acceso e inclusión en el Registro Único (CadÚnico) ^( 28 , 30 , 32 , 47 )^ por la oferta calificada de servicios, programas, proyectos y beneficios de socioasistenciales del gobierno federal a las personas con tuberculosis en situación de riesgo y vulnerabilidad ^( 6 , 28 , 31 , 45 , 47 )^ .
		Reconocimiento de las personas con tuberculosis como criterio de elegibilidad para los programas y servicios de asistencia social ^( 29 , 32 )^ .
		Uso del CRAS ^||^ , CREAS ^¶^ , Centro Pop ^‡^ , Unidades de Acogida, entre otros, como espacios para el desarrollo de actividades dirigidas a la protección social de las personas afectadas por la tuberculosis ^( 28 - 29 , 47 )^ .
*Derecho a la asistencia social*	BPC**	Pago de un salario mínimo mensual a las personas con discapacidad de cualquier edad o personas mayores, de 65 años o más, que no puedan sustentarse por sí mismas ni ser sustentadas por su familia ^( 30 , 32 )^ .
	Beneficios eventuales	Acceso a beneficios financieros, de carácter complementario y temporal, en situaciones de nacimiento, muerte, vulnerabilidad temporal y calamidad pública ^( 30 , 32 )^ .
	Documentación civil	Acceso a la documentación civil básica ^( 29 - 30 , 47 )^ .
	Programas de transferencia de ingresos	Reconocimiento de las personas con tuberculosis como criterio de elegibilidad para los programas de transferencia de ingresos ^( 29 )^ , como el PBF ^††^ u otros programas estatales y municipales a los que también se puede recurrir ^( 30 )^ .
	Servicios de acogida del SUAS ^†^	Reconocimiento de las personas con tuberculosis como criterio de elegibilidad para el acceso a los servicios de acogida ^( 29 )^ .
		Garantía de acceso a los servicios de acogida a las personas con tuberculosis que no cuenten con vivienda, evaluando la posibilidad de permanencia durante al menos seis meses de tratamiento ^( 29 - 30 , 47 )^ .
		Creación y acceso a Casas de Acogida para personas afectadas por tuberculosis, con vulnerabilidad social, que no cuentan con apoyo familiar para los cuidados de salud ^( 24 - 25 )^ .
*Derecho a la Seguridad Social*	Jubilación por incapacidad permanente	Exención del periodo de carencia para los trabajadores en plantilla o autónomos que coticen al INSS ^‡‡^ para la concesión de jubilación por incapacidad permanente cuando la incapacidad laboral se deba a tuberculosis activa ^( 19 , 30 - 32 , 35 , 43 )^ .
	Subsidio por incapacidad temporal (pensión por enfermedad)	Exención del periodo de carencia para trabajadores en plantilla o autónomos que coticen al INSS ^‡‡^ para la concesión de subsidios por incapacidad temporal, es decir, si no pueden trabajar durante más de 15 días consecutivos en virtud de enfermedad debida a tuberculosis activa ^( 30 - 32 , 35 , 43 )^ .
	Exención del impuesto sobre la renta	Exención sobre los ingresos relativos a la jubilación, pensión o retiro, incluida la complementación recibida de una entidad privada y la pensión alimenticia para personas que padecen enfermedades graves, como la tuberculosis activa ^( 27 , 29 - 30 , 34 , 46 )^ .
	Transacciones financieras del PIS/PASEP ^§§^	Posibilidad de realizar transacciones en la cuenta PIS/PASEP ^§§^ por parte de titulares o dependientes con tuberculosis activa ^( 21 )^ .
*Reparto de responsabilidades*	Coordinación entre SUAS ^†^ y SUS*	Actuación del SUAS ^†^ en articulación con el SUS* para el afrontamiento de la tuberculosis, mediante la integración entre la Red Socioasistencial y la RAS ^||||(6,20,24,28-30,33,38-39,41,45,47)^ .
		Orientación por parte de los profesionales del SUAS ^†^ sobre la vacuna BCG ^¶¶^ , la importancia de que las personas con sospecha de tuberculosis acudan al servicio de salud más cercano y sobre el apoyo para completar el tratamiento ^( 29 )^ .
		Establecimiento de mecanismos de coordinación y reparto de cuidados entre los servicios de asistencia social de Protección Social Básica y Protección Social Especial de media y alta complejidad con los servicios de la RAS ^||||(28-29,47)^ .
	Acciones intersectoriales e interministeriales	Coordinación entre el Ministerio de Ciudadanía y el Ministerio de Salud para afrontar la tuberculosis, especialmente entre personas en situación de vulnerabilidad social ^( 33 , 41 , 45 , 47 )^ .
		Coordinación interministerial ^( 6 , 33 , 35 )^ y actuación del CIEDDS*** en el ámbito del Ministerio de Salud ^( 40 , 42 , 44 )^ .
		Coordinaciones intersectoriales con áreas estratégicas como Atención Primaria, Salud Indígena, Salud Mental, Salud Ocupacional y actuación multisectorial por medio de la Red Brasileña de Comités para el Control de la Tuberculosis, la Red Brasileña de Enfermería para el Fin de la Tuberculosis como Problema de Salud Pública, la Alianza Brasileña de Lucha contra la Tuberculosis y el Frente Parlamentario de Lucha contra la Tuberculosis ^( 6 )^ .
	Celeridad de la justicia	Prioridad en la tramitación de procesos judiciales y administrativos en los que figuren como parte o interesado una persona enferma de tuberculosis ^( 26 , 30 )^ .

*SUS = Sistema Único de Salud; ^†^SUAS = Sistema Único de Asistencia Social; ^‡^Centro Pop = Centro de Referencia Especializado para la Población en Situación de Calle; ^§^FGTS = Fondo de Garantía por Tiempo de Servicio; ^||^CRAS = Centro de Referencia de Asistencia Social; ^¶^CREAS = Centro de Referencia Especializado de Asistencia Social; **BPC = Beneficio de Prestación Continua; ^††^PBF = Programa Bolsa Familia; ^‡‡^INSS = Instituto Nacional de la Seguridad Social; ^§§^PIS/PASEP = Programa de Integración Social y Programa de Formación de Patrimonio del Servidor Público; ^||||^RAS = Red de Atención a la Salud; ^¶¶^BCG = Bacilo de Calmette y Guérin; ***CIEDDS = Comité Interministerial para la Eliminación de la Tuberculosis y de Otras Enfermedades Determinadas Socialmente

## Discusión

En el contexto brasileño, considerando la importancia de que las personas afectadas por TB sean consideradas como sujetos en su integralidad, las políticas de protección social tienen la perspectiva de promover el acceso de la población a sus derechos fundamentales, con el fin de garantizar el ejercicio de la ciudadanía, la atención de las necesidades básicas y la promoción de la dignidad humana^(31)^. Además, materializan la importancia legal, ética y moral ante la eficacia de su respuesta en los desenlaces del tratamiento de la TB y el alivio del sufrimiento entre las personas y familias afectadas por la enfermedad^(48)^.

Un estudio señala que la disponibilidad y la distribución gratuita del medicamento antituberculosis no son suficientes para la continuidad del tratamiento o la curación de la TB, ya que la adhesión al tratamiento no se reduce a una voluntad de carácter exclusivamente individual, sino que se asocia a otras dimensiones transversales a los procesos de producción y reproducción social^(49)^. Esto sugiere un enfoque coordinado que incluya no solo los servicios de salud, sino también la coordinación intra e intersectorial basada en un sólido sistema de protección social.

En ese sentido, es necesario repensar políticas públicas que representen agendas específicas y comprender lo que traducen, su construcción histórica y las relaciones de poder establecidas, que tomen en cuenta los deseos y las necesidades de la población. Esto se debe a que las instituciones de poder no se preocupan realmente por las políticas como instrumento social, salvo aquellas de naturaleza meramente asistencial que solo mitigan las desigualdades sociales^(50)^.

Tras la promulgación de la Constitución Federal de 1988, que presenta el sistema de seguridad social brasileño, la intersección entre salud y derechos humanos fue revitalizada con la inclusión de la salud como “*derecho de todos y deber del Estado”* ^(15)^. Con base en ese contexto, se identificó la construcción de un camino heterogéneo de las políticas públicas de protección social para las personas afectadas por TB en el país, marcado por diferentes períodos políticos, sociales e ideológicos, con la reanudación de la visibilidad recién después de 21 años de la promulgación de la Carta Magna brasileña y del cambio de la legislación en materia de declaración del impuesto sobre la renta^(27)^, con una ley destinada a agilizar los procesos judiciales y administrativos, que incluyó a las personas con TB^(26)^.

Posteriormente a ese tímido escenario, el año 2019 marcó un hito importante para la discusión de la TB como un problema de salud pública cuyo afrontamiento trasciende el ámbito de la salud. Ese año se publicaron dos documentos normativos: el Manual de Recomendaciones para el Control de la Tuberculosis en Brasil^(32)^ y la Instrucción Operativa Conjunta n.º 1/2019^(47)^, que apoyan y orientan la mayoría de las políticas públicas orientadas a la protección social identificadas en este estudio, ya que presentan estrategias para fortalecer la articulación intra e intersectorial para garantizar los derechos humanos y la ciudadanía en las acciones de prevención y cuidado de la TB^(32,47)^.

A partir de ahí, para la alineación con el Pilar 2 de la estrategia *The End TB*, centrado en las políticas audaces y los sistemas de apoyo, la TB comienza a ser incluida con mayor fuerza en las agendas políticas de las esferas gubernamentales brasileñas, mediante documentos normativos articulados con los poderes ejecutivo y legislativo, así como en las agendas de trabajo de la salud, seguridad y asistencia social que posibilitan la protección social a las personas afectadas por TB^(32)^. Desde esta perspectiva, las acciones de prevención y cuidado de la TB deben guiarse por el afrontamiento de las desigualdades sociales e inequidades de salud que afectan a las personas que padecen de la enfermedad y sus familiares, buscando romper los ciclos de pobreza y miseria, y buscando también la sostenibilidad en la generación de empleo e ingresos^(2)^.

Se estima que las enfermedades y muertes por TB podrían causar un grave impacto en la economía mundial de alrededor de un billón de dólares entre el 2015 y el 2030^(51)^. Por otra parte, un estudio sostiene que las estrategias de protección social de base gubernamental, con un compromiso político sólido y recursos financieros adecuados, tienen un mayor potencial para lograr la meta global de un 85% de éxito en el tratamiento de la TB^(52)^. Para ello, una de las cuestiones primordiales se refiere a garantizar la inversión pública, de modo que tanto el presupuesto centralizado del Ministerio de Salud como los recursos descentralizados por medio de fondos estatales o municipales se amplíen a las tres esferas de la gestión brasileña^(42)^.

Este escenario solo es posible por medio de políticas públicas que interfieren en la producción de salud en el territorio, como las centradas en la seguridad alimentaria y nutricional, vivienda, saneamiento, agua, empleo, ingresos, educación, transporte, entre otras^(40)^, redescubriendo el concepto ampliado de salud recomendado en la Constitución Federal y en la Ley Orgánica de Salud^(17)^.

También se pudo identificar el protagonismo del SUS en acciones de prevención y cuidado integral a las personas afectadas por TB, considerando la transversalidad del derecho a la salud. Sin embargo, a pesar de que el SUS ofrece tratamiento universal y gratuito^(32)^, se entiende que la TB causa pérdidas y empeora la situación socioeconómica, lo que puede desestabilizar la dinámica familiar al generar gastos adicionales con alimentación, transporte, otros medicamentos y exámenes, por ejemplo^(39,47)^.

Estos gastos, ya sean directos o indirectos, cuando superan el 20% de los ingresos familiares anuales, se consideran costos catastróficos generados por la enfermedad de TB^(53)^ e impactan directamente en la adhesión y el desenlace del tratamiento de la enfermedad, además de provocar consecuencias sociales^(54)^. Para mitigar dichos costos o incluso, de manera audaz, lograr la meta de que ninguna persona con TB tenga que soportar costos catastróficos o repercusiones sociales debido a la enfermedad^(3)^, es necesario garantizar que las personas con TB y las familias afectadas tengan acceso a intervenciones de protección social contra los riesgos financieros derivados del tratamiento^(55)^.

Una de las propuestas para esta situación es el Proyecto de Ley (PL) n.º 6.991, de 2013, que tiene como objetivo garantizar apoyo económico a las familias en situación de pobreza que tengan, entre sus miembros, personas afectadas por la TB, mediante el pago de un beneficio financiero equivalente a medio salario mínimo durante la duración del tratamiento de la TB^(56)^. Este PL, incluso después de 10 años de su elaboración, aún se encuentra en tramitación en la Cámara de Diputados, lo que puede indicar una falta de prioridad para la aprobación de leyes que aborden la transferencia condicionada de ingresos para afrontar enfermedades determinadas por la pobreza, como la TB.

Planificar e implementar políticas de salud en coherencia con las políticas sociales puede resultar muy eficaz en países con una alta carga de TB. Un ejemplo exitoso de esto en la Región de las Américas es Argentina, que si bien no figura en la lista de 30 países en esta situación, implementó la política de transferencia condicionada de ingresos para las personas afectadas por TB mediante el Decreto n.º 170/91 de la Ley 10.436 de financiamiento estatal para pagar un salario mínimo a todas las personas elegibles vinculadas al Programa Provincial de Control de la TB. Esta ley de protección económica fue fundamental para motivar y monitorear los casos de difícil manejo, aumentando la adhesión al tratamiento de la TB^(57-58)^.

Cabe resaltar que los derechos de las personas afectadas por la TB son los mismos que los de la población en su conjunto, sin distinción, aunque existen situaciones y condiciones para el acceso a derechos específicos en el ámbito de la salud, la asistencia y la seguridad social. En ese sentido, este estudio identificó un conjunto de medidas que abarcaron políticas públicas orientadas a la inclusión social, al afrontamiento de la pobreza y a la garantía de acceso a los derechos sociales, la seguridad alimentaria y nutricional, el transporte, el trabajo, la vivienda y los beneficios de seguridad social de las personas afectadas por la TB.

Algunas de estas estrategias de protección social están más centradas en el tratamiento de la TB y se caracterizan como “acciones específicas para la TB”, que benefician a las personas afectadas por la enfermedad y sus familias y se incorporan a los programas de tratamiento de la TB existentes. Otras son parte de una concepción ampliada de seguridad social, que tiene un gran potencial para modificar las condiciones estructurales de la sociedad, al fortalecer la resiliencia económica, aliviar la pobreza y actuar sobre otros determinantes sociales intrínsecos a la enfermedad y la continuidad de la cadena de transmisión de la TB^(59)^.

Un estudio realizado en Brasil identificó las potencialidades en la implementación de acciones específicas para la TB, a ejemplo de la provisión de bonos de alimentación a personas afectadas por la TB, lo que aumentó la tasa de curación en un 13% en comparación con el grupo sin dicha intervención^(60)^. En cuanto a las acciones sensibles para la TB, los estudios presentaron uno de los mayores ejemplos brasileños: el Programa Bolsa Familia (PBF), que, mediante transferencias condicionadas de ingresos, fue responsable de una tasa de curación del 7,6%^(61)^ y 8%^(62)^ mayor y una pérdida de seguimiento un 7% menor en grupos beneficiarios del PBF con TB^(61)^.

Sin embargo, es importante comprender los desafíos que permean la consolidación de estas políticas en el afrontamiento de la TB. Para garantizar los derechos analizados en este estudio, el acceso a las políticas de protección social estuvo sujeto a condicionalidades, como la necesidad de documentación civil, vinculación al Registro Único para acceder al SUAS o cotizaciones obligatorias al INSS para acceder a los beneficios de la seguridad social, lo que puede generar barreras a la consecución de derechos, sobre todo para poblaciones en situación de mayor vulnerabilidad social, como las personas en situación de calle.

Esto se debe a que, además de que esta población es considerada la más vulnerable a enfermar de TB, con un riesgo 56 veces mayor en comparación con la población general del país^(30)^, es evidente la discriminación e invisibilidad de la que son objeto estas personas, ya sea por la falta de documentos, de residencia, por estereotipo o por el consumo de drogas, lo que refuerza la desasistencia, las privaciones y la restricción de derechos, de la ciudadanía y de la propia condición humana^(63)^.

Durante la pandemia de COVID-19, hubo indicios de que estas cuestiones se intensificaron aún más, considerando la superposición de vulnerabilidades sociales y programáticas y el propio impacto de las medidas restrictivas en la sociedad y en las acciones de prevención y cuidado de la TB^(64-65)^. Para afrontar las barreras de acceso a los derechos y acciones de cuidado integral de salud, durante este período se elaboraron algunos documentos normativos orientados a la protección social de estas personas, con énfasis en la articulación y en el reparto del cuidado entre los equipos del Centro Pop y de los Consultorios en la Calle^(37,39)^.

Las normativas destinadas a combatir el estigma y la discriminación de la TB en el país siguen un movimiento de combate a las concepciones que marcaron la vivencia de la TB en el pasado y que hasta el día de hoy imponen restricciones y obstáculos al tratamiento, una vez que complican la trayectoria de cuidado y marginan a las personas afectadas por la enfermedad^(30,47)^. Así, los documentos que presentan medidas encaminadas a este tipo de combate permiten rescatar el derecho a situaciones que no sean humillantes, degradantes u ofensivas a la dignidad humana^(48)^.

La actuación intersectorial, como entre el SUS y el SUAS, y la coordinación interministerial por medio del Comité Interministerial para la Eliminación de la Tuberculosis y de Otras Enfermedades Determinadas Socialmente (CIEDDS)^(44)^ avanzan desde la perspectiva de la priorización de estrategias que promuevan la protección social. Esta priorización se puede lograr mediante la sinergia entre áreas ministeriales estratégicas para afrontar los determinantes sociales de la TB en los ámbitos de asistencia social, justicia y seguridad pública, trabajo e ingresos, derechos humanos, igualdad racial, pueblos originarios, educación y ciudadanía^(40,42,44)^. Además, cabe resaltar la importancia de la responsabilidad social y de la participación de áreas como la enfermería, que pueden contribuir sustancialmente a la reducción de las desigualdades sociales y sanitarias subyacentes de diferentes contextos comunitarios^(66)^.

Como limitaciones de este estudio, cabe resaltar la actualización de las plataformas de búsqueda, que, si bien han recuperado un volumen robusto de información, presentan debilidades en cuanto a la disponibilidad de materiales, y algunos documentos normativos pueden no haber sido identificados porque no están disponibles electrónicamente. Además, las discusiones planteadas tienen un contenido crítico-reflexivo por parte de los investigadores y pueden no haber abarcado otras interpretaciones que también se aplican al ámbito de disputa de las políticas públicas brasileñas, con el recorte analítico de la protección social de las personas afectadas por la TB.

Finalmente, los resultados de esta investigación documental avanzan en el conocimiento científico en el campo de la salud pública al ampliar la comprensión de las complejas interacciones entre el derecho a la salud, los derechos sociales y la justicia social en el contexto de afrontamiento de la TB, proporcionando aportes para guiar la formulación e implementación de políticas públicas más eficaces e inclusivas destinadas a reducir las desigualdades sociales y sanitarias que afectan fuertemente a las personas que padecen de TB y a sus familiares.

## Conclusión

Este estudio identificó y analizó documentos normativos que buscan garantizar el derecho a la protección social por medio de políticas públicas de afrontamiento a la TB que cubran el derecho a la salud, a la asistencia y a la seguridad social, así como acciones que impliquen el reparto de responsabilidades para el cumplimiento de estos derechos.

Dichas políticas se consideran recientes en la historia del Estado Democrático de Derecho y aún tienen un largo camino que recorrer hacia un enfoque integral, centrado en las personas afectadas por la TB y que, de hecho, se basen en derechos para la prevención, el cuidado y el apoyo mediante articulaciones intersectoriales e interministeriales, con fuerte actuación del SUS y del SUAS.

Sin embargo, los desafíos que impone la dificultad o la falta de acceso a estos derechos, asociados a otras situaciones que potencian la vulnerabilidad social, hacen que las personas afectadas por TB permanezcan socialmente desprotegidas. En ese sentido, cabe reafirmar el papel y el deber del Estado en garantizar la protección social mediante políticas públicas que promuevan la vida, la ciudadanía, los derechos humanos y la justicia social.
